# Regulatory T cells are associated with the tumor immune microenvironment and immunotherapy response in triple-negative breast cancer

**DOI:** 10.3389/fimmu.2023.1263537

**Published:** 2023-09-12

**Authors:** Pengfei Huang, Xinyue Zhou, Minying Zheng, Yongjun Yu, Gongsheng Jin, Shiwu Zhang

**Affiliations:** ^1^ Department of Surgical Oncology, The First Affiliated Hospital of Bengbu Medical College, Bengbu, Anhui, China; ^2^ Graduate School, Tianjin Medical University, Tianjin, China; ^3^ Department of Pathology, Tianjin Union Medical Center, Tianjin, China

**Keywords:** triple-negative breast cancer, regulatory T cells, tumor microenvironment, single-cell RNA-sequencing, prognostic signature

## Abstract

**Introduction:**

Triple-negative breast cancer (TNBC) is the most aggressive subtype of breast cancer with a high risk of distant metastasis, an extremely poor prognosis, and a high risk of death. Regulatory T cells (Tregs) contribute to the formation of a tumor immunosuppressive microenvironment, which plays an important role in the progression and treatment resistance of TNBC.

**Methods:**

A public single-cell sequencing dataset demonstrated increased infiltration of Tregs in TNBC tissues relative to normal breast tissue. Weighted gene co-expression network analysis was used to identify Treg infiltration-related modules for METABRIC TNBC samples. Subsequently, we obtained two Treg infiltration-associated clusters of TNBC by applying consensus clustering and further constructed a prognostic model based on this Treg infiltration-associated gene module. The ability of the selected gene in the prognostic model, thymidine kinase-1 (TK1), to promote the progression of TNBC was evaluated *in vitro*.

**Results:**

We concluded that two Treg infiltration-associated clusters had different prognoses and sensitivities to drugs commonly used in breast cancer treatment, and multi-omics analysis revealed that the two clusters had different copy number variations of key tumor progression genes. The 7-gene risk score based on TNBC Treg infiltration was a reliable prognostic indicator both in the training and validation cohorts. Moreover, patients with TNBC with high Treg infiltration-related scores lacked the activation of immune activation pathways and exhibited resistance to anti-PD1 immunotherapy. Knocking down TK1 led to impaired proliferation, migration, and invasion of TNBC cells *in vitro*. In addition, specimens from patients with TNBC with high TK1 expression showed significantly higher Treg infiltration in tumors. Results of spatial transcriptome analysis showed that TK1 positive cells mainly localize in tumor area, and Treg cell infiltration in TNBC tissues was associated with high expression of TK1. Pan-cancer analysis also demonstrated that TK1 is associated with poor prognosis and activation of proliferation pathways in multiple cancers.

**Discussion:**

We established a prognostic model related to Treg infiltration and this model can be used to establish a clinically relevant classification of TNBC progression. Additionally, our work revealed the underestimable potential of TK1 as a tumor biomarker and immunotherapeutic target.

## Introduction

Breast cancer is the most frequently diagnosed type of cancer in women and is the primary cause of cancer-related deaths (1). Triple-negative breast cancer (TNBC) accounts for 10–20% of patients diagnosed with breast cancer each year and generally affects young females, particularly those of African descent (2). Breast cancers are classified as triple negative, luminal (ER/PR receptor positive), and HER2/neu based on gene expression profiles. This classification scheme has been documented to have prognostic significance and treatment response implications (3–6). TNBC refers to breast cancer that is negative for estrogen receptor (ER), progesterone receptor (PR), and human epidermal growth factor receptor expression (HER2) (5). As the most lethal breast cancer subtype, TNBC is a highly aggressive endocrine malignancy associated with high rates of recurrence and distant metastasis (7). To date, there are no specific treatment guidelines for TNBC. Despite advances in chemotherapy and neoadjuvant immunotherapy to improve patient prognoses, many patients continue to develop chemoresistance (8). The possibility of chemoresistance in patients with TNBC varies. One speculation is that it is closely associated with the tumor microenvironment (TME).

The TME is crucial to the development and progression of cancer. In contrast to other subtypes, TNBC features a distinctive TME to stimulate progression (9). The role of the TME in anti-tumor responses is reflected in its ability to induce proliferation and angiogenesis, inhibit apoptosis, suppress the immune system, and evade immune surveillance (10). The composition of the TME includes transformed extracellular matrix (ECM), soluble cytokines, immunosuppressive cells, epigenetic modifications, and reprogrammed fibroblasts (9). Cancer-associated fibroblasts play an important role in the TME. Biglycan, a cancer-associated fibroblast-specific secreted factor, can be applicable in clinical practice and serve as a therapeutic target for immunotherapy resistance in breast cancer (11). Tumor-associated macrophages (TAM) have an essential role in the progression of TNBC, including driving the aggressive cellular phenotype in various cancers. Macrophages are functionally malleable and can switch their polarization state from M1 to M2 to adapt to varying physiological conditions (12). The TME creates a favorable environment for cancer cells to interact with the surrounding endothelial cells, immune cells, and fibroblasts (13).

As a specialized subset of T cells, T-regulatory cells (Tregs) can suppress anti-tumor immune responses and protect against autoimmunity by restraining T-cell proliferation and cytokine production (14). TME-infiltrating Tregs can create an immunosuppressive environment by activating immune-inhibitory and pro-tumorigenic signaling, which contributes to reducing the impact on chemotherapy and radiotherapy responses (15). Breast cancer cells secrete chemokines that bind to Treg surface receptors to trigger Treg expansion (16). The potential role of Tregs in the TME and potential therapeutic targets of Treg cells may provide novel interventions for tumor immunotherapy in TNBC.

**Figure 1:**
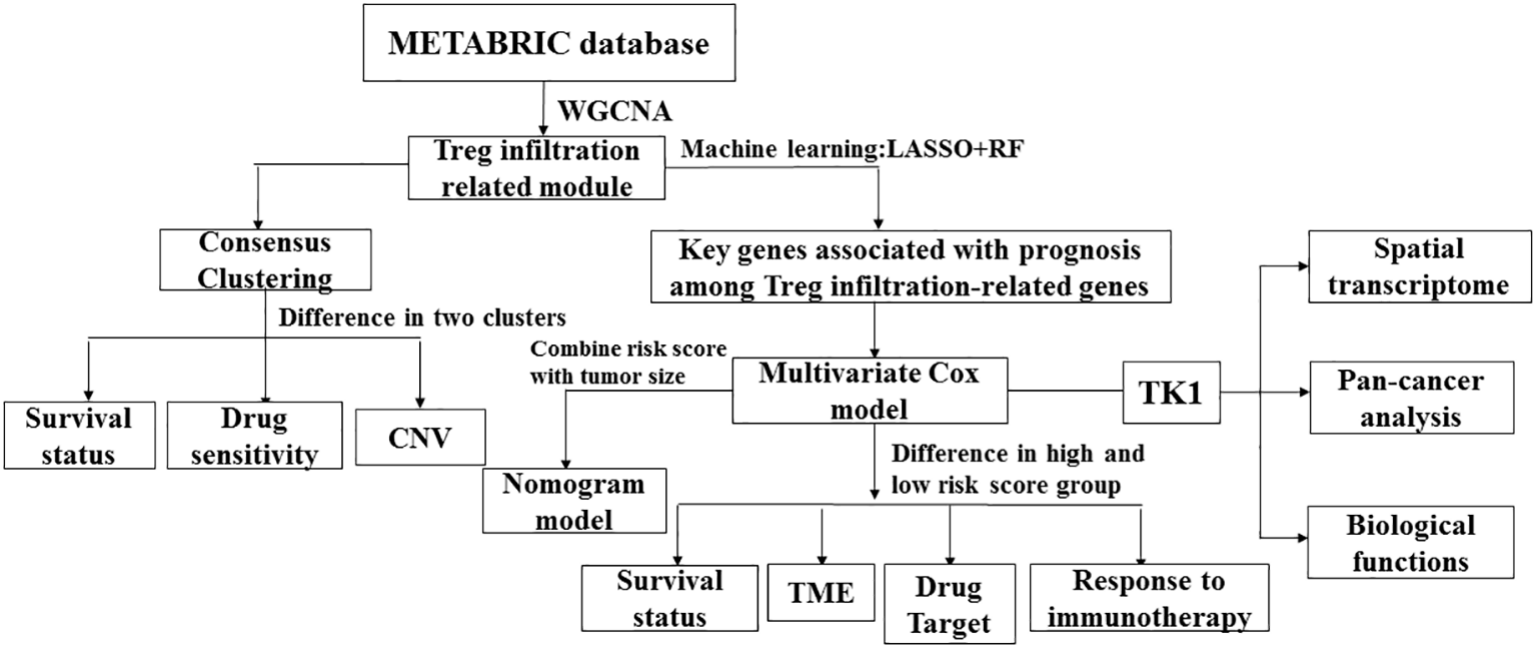
The workflow of this study.

Weighted gene co-expression network analysis was utilized to identify modules related to Treg infiltration in METABRIC TNBC samples. Subsequently, a prognostic model was developed based on the modules associated with Treg infiltration. Patients in the METABRIC cohort were divided into high- and low-risk groups based on the risk score associated with Treg infiltration. In this study, we conclude that one of the main reasons for the lower overall survival (OS) of patients in the high-risk group is the extensive infiltration of Tregs and the disturbance of immune response pathways. Thymidine kinase-1 (TK1), lysyl oxidase (LOX), lysine demethylase 5B (KDM5B), proteasome 26S subunit non-ATPase 4 (PSMD4), and nuclear factor erythroid 2-like 3 (NFE2L3) genes are implicated in relapse and poor prognosis of patients with breast cancer. Among these genes, TK1 was found to have significant prognostic value in TNBC. As a cell cycle-dependent kinase, TK1 can regulate cellular proliferation through restoration of the nucleotide thymidine in the DNA repair pathway (17). There is much research on the role of TK1 as a diagnostic biomarker for several cancer types including chronic lymphocytic leukemia (CLL), glioma, and others (18). Elevated serum TK1 levels have been shown to predict CLL disease progression and enable medical workers to identify patients with CLL at high risk of rapid progression and early stage (19). Single-cell functional analysis revealed that TK1 expression positively correlated with the proliferation, cell cycle, DNA repair, DNA damage, and epithelial-mesenchymal transition of glioma cells. TK1 is expressed at high levels in gliomas, and the general survival is worse in patients with high TK1 expression (20). Serum TK1 concentration also indicates the prognostic potential of patients with malignant tumors. TK1 expression is barely detectable in normal serum but is variably high in malignant tumors, depending on the type, stage, growth rate, and treatment of malignant tumors (21–23). Overall, Treg infiltration-associated gene module can be used to establish a clinically relevant classification of TNBC progression. Our work revealed the underestimable potential of TK1 as a tumor biomarker and immunotherapeutic target. The overall design of this study is illustrated in **Figure 1**.

## Methods

### Dataset source and data pre-processing

Gene expression and clinical data of 1,904 patients with breast cancer from the Molecular Taxonomy of Breast Cancer International Consortium (METABRIC) dataset were downloaded from cBioPortal (https://www.cbioportal.org/). Additionally, clinical and transcriptome data of two other breast cancer datasets were downloaded from the Gene Expression Omnibus (GEO), including GSE58812 and SCAN-B. After excluding patients who were positive for ER, PR, and HER2, 298 patients in the METABRIC dataset, 333 patients in SCAN-B, and 107 patients in GSE58812 were included for further analysis. For the single-cell RNA sequencing (scRNA-seq) dataset, GSE161529 raw data were downloaded from GEO. scRNA-seq data processing was performed using the R ‘Seurat’ package, as described in the tutorial. Briefly, cells with gene expression <300 or >6500 and mitochondrial gene expression >10% were excluded. It applied the SCTransform function to normalize and scale the raw counts before performing a principal component analysis (PCA). The R “Harmony” package was used to remove batch effects from isolated scRNA-seq raw data. Using unsupervised cluster analysis and unified manifold approximation and projection (UMAP), we identified distinct clusters of cells in each scRNA-seq dataset. Each cell cluster was then annotated based on known cell type marker genes.

### Weighted gene co-expression network analysis (WGCNA)

Gene co-expression networks were constructed based on METABRIC transcriptome data using the R ‘WGCNA’ package. We calculated Pearson’s correlation coefficient between each pair of genes to obtain a similarity matrix. ‘WGCNA’ has a power function that can convert the similarity matrix into an adjacency matrix. Among all soft thresholds (β) with R2 > 0.9, we chose the automatic value of β (β = 5) returned by the WGCNA pick soft-threshold function. According to the recommendations of the ‘WGCNA’ tutorial, the network merge height was chosen to be 0.25. Other WGCNA parameters used the default settings for further analysis.

### Gene set variation analysis (GSVA) and estimation of immune cell infiltration

GSVA was performed using the R ‘GSVA’ package 16 to calculate related gene set enrichment scores. High and low GSVA scores were used to compare the enrichment of up- or downregulated associated pathways in the high-risk score group relative to the low-risk score group. All GSVA gene sets were downloaded from MSigDB v7.4. Immune infiltration in the METABRIC and GEO cohorts was quantified using the CIBERSORT algorithm based on normalized expression data.

### Treg cell infiltration-related cluster acquisition

We selected a module associated with Treg cell infiltration and then performed univariate Cox regression analysis on the genes in this module. The 22 genes (*P* < 0.05) associated with survival in univariate analysis were then input into the R ‘ConsensusClusterPlus’ package for consensus clustering of METABRIC patients. According to the results of the cluster consensus value and cumulative distribution function, the optimal K value was determined to be 2.

### Development of the Treg-related prognostic model

A Treg-related prognostic model was established based on three TNBC cohorts, including METABRIC as the training dataset and another two cohorts as validation datasets (SCAN-B and GSE58812). The 22 prognosis-related genes described above were further screened for key genes related to Treg cell infiltration and patient prognosis using two machine learning methods, least absolute shrinkage and selection operator (LASSO) logistic regression and random forest. First, 17 prognostic genes were selected using the R ‘glmnet’ package to perform LASSO regression with 10-fold cross-validation. Subsequently, 13 prognostic-related features were screened using the random forest algorithm in the R ‘randomForestSRC’ package. Finally, seven common genes (TK1, SRM, PSMD4, NFE2L3, LOX, KDM5B, and RITA1) were obtained to build the multivariate Cox regression models (both using stepwise regression). The risk score was calculated as follows:0.236∗(TK1 expression) + 0.321∗(SRM expression)-0.512∗(PSMD4 expression)-0.124∗(NFE2L3 expression)+0.292∗(LOX expression)+0.463∗(KDM5B expression)+0.457∗(RITA1 expression). The same model score threshold was used to calculate the risk score in the METABRIC and validation cohorts. Patients were divided into low- and high-risk groups according to the median risk score cutoff, and differences in OS were compared using the R ‘survival’ package. We calculated the area under the curve (AUC) using the R ‘timeROC’ package to assess prognostic model accuracy.

### Nomogram construction

We created a nomogram including the risk score and tumor size using the regplot function in the R ‘rms’ package. A receiver operating characteristic (ROC) curve was plotted to check the accuracy of our predictive model. We also plotted calibration and decision curve analysis (DCA) curves to illustrate the discrepancy between our model and actual observed patient survival.

### Chemotherapeutic response prediction

The chemotherapeutic response for each cluster was predicted based on the largest publicly available pharmacogenomics database (Genomics of Drug Sensitivity in Cancer [GDSC], https://www.cancerrxgene.org/). Four chemotherapeutic drugs commonly used in the treatment of TNBC, including Doxorubicin, Gefitinib, cisplatin, and gemcitabine, were selected for further analysis. The prediction process was conducted using the R ‘pRRophetic’ package. The half-maximal inhibitory concentration (IC50) of samples was estimated using ridge regression, and prediction accuracy was assessed by 10-fold cross-validation based on the GDSC training set. All parameters were set to default values, except for tissue type as ‘breast’.

### Spatial transcriptomics sequencing data analysis

The publicly available Spatial Transcriptome Dataset used in this study is available from the Gene Expression Omnibus (accession numbers GSE210616). All details of spatial transcriptomics data processing and analysis performed in this work are referred to the Seurat website tutorial (https://satijalab.org/seurat/articles/spatial_vignette.html). To integrate the data, the spatial transcriptome data were pre-processed by the “SCTransform” function and PCA analysis. Additionally, the robust cell type decomposition (RCTD) approach (https://github.com/dmcable/spacexr) was used to infer the cell type composition at each spatial location. A published scRNA-seq data (GSE176078) was used as a reference panel for RCTD fitting.

### Pan-cancer analysis

The normalized mRNA expression and clinical information of TCGA pan-cancer cohorts (**Supplementary Table 1**) were download from the UCSC Xena Browser (https://xenabrowser.net/datapages/).

### Data and code availability

Public data used in this work can be download from the UCSC Xena (xenabrowser.net/datapages/) database and Gene Expression Omnibus (GEO, http://www.ncbi.nlm.nih.gov/geo/). The essential analysis script is available from GitHub at https://github.com/TNBC222/TNBC_Tregs and 10.6084/m9.figshare.23750538.

### Cell culture

Two TNBC cell lines, BT-549 and MDA-MB-231, were obtained from the American Type Culture Collection (ATCC; Manassas, VA, USA). BT-549 cells were cultured in Roswell Park Memorial Institute-1640 medium (1×) (Gibco, Thermo Fisher Scientific, Suzhou, China) supplemented with 10% fetal bovine serum (FBS; Gibco, Life Technologies, New Zealand), 1% penicillin-streptomycin (Gibco, Life Technologies, USA), and insulin. MDA-MB-231 cells were maintained in Dulbecco’s modified Eagle’s medium (DMEM) supplemented with 10% FBS and 1% penicillin-streptomycin. The cells were incubated at 37°C in a humidified atmosphere containing 5% CO_2_.

### Transient small interfering RNA (siRNA) transfection

TK1 was knocked down using transient small interfering RNA (siRNA) transfection. siRNA oligonucleotides were synthesized by Gene-pharma (Shanghai, China), including three siRNA interference sequences (S248, S363, and S683), one positive control sequence (GAPDH), one negative control sequence (NC), and one mock control sequence (MC) with only transfection reagents. TK1-248 and TK1-683 were demonstrated to have significant inhibitory effects on TK1 expression and were used in this study.

### Western blot analysis

The control and TK1-siRNA (TK1i)-transfected BT-549 and MDA-MB-231 cells were collected and lysed. The protein samples were loaded onto 10% sodium dodecyl sulfate-polyacrylamide gels and separated by electrophoresis. Subsequently, the separated proteins were transferred to polyvinylidene fluoride (PVDF) membranes. The PVDF membranes were then soaked in 5% skim milk for approximately 1 h at room temperature. The membranes were incubated with the first antibodies at 4°C overnight (**Supplementary Table 2**). Then, the membranes were further incubated with the secondary antibody at room temperature for at least 1 h the next day. β-actin (Sigma-Aldrich) was used as a loading control. All western blotting results were duplicated at least three times. Protein expression was detected using a Bio-Rad imaging system and ImageJ software.

### Plate colony formation assay

The proliferative ability of cells was evaluated using a plate colony formation assay. Three groups of both TNBC models were cultured with 30, 60, or 120 cells in 12-well plates at 37°C with 5% CO_2_ for 2 weeks. When visible cell clones appeared in the 12-well plate, incubation was terminated, the cells were fixed with absolute methanol for 30 min, and the cell colonies were stained with 0.1% crystal violet for 30 min. The number of cell colonies was counted under a microscope, and a single colony was defined as a cluster containing at least 50 cells.

### Wound-healing assay

A wound-healing assay was performed to evaluate the migration ability of different cell groups (TK1 knockdown and control). Each cell line had one control group and two knockdown groups. The cells were cultured in a 6-well plate. When confluency reached approximately 95%, wound tracks were created at the back of the plate by scraping the cell monolayer with sterile pipette tips. Detached cells were gently removed by washing three times with PBS. Subsequently, cells were cultured in serum-free culture medium at 37°C with 5% CO_2_ and photographed at 0 h and 24 h under a microscope. The scratched area was measured using ImageJ software.

### Cell counting kit-8 (CCK8) assay

The viability of both BT-549 and MDA-MB-231 cells was assessed by the CCK8 assay. Cells were seeded in a 96-well plate at an appropriate density (2x10^3^ cells per well) and were incubated for different times (0h,24h,48h,72h,96h). CCK-8 solution (10%; Dojndo, Japan) was added to each well and the plate was incubated for 1–4 h. The absorbance of each well was measured using a microplate reader.

### Transwell assay

The migration ability was measured via transwell migration assay using cell culture inserts. Cells (5×10^4^ cells per insert) in 200 μL medium without FBS were seeded in the upper chamber while 600 µL medium containing 20% FBS was added to the lower chamber. Then cells were cultured in a CO_2_ incubator for 24h. After removing the medium, the cell culture inserts were fixed in methanol for 30 min and stained with 0.1% crystal violet for 30 min. Cells were counted from at least 5 different fields by ImageJ software. Independent experiments were performed three times.

### Human TNBC samples

Paraffin-embedded TNBC (n=31) tissue samples were collected from the Department of Pathology in Tianjin Union Medical Center. The Hospital Review Board of the Tianjin Union Medical Center approved this study and the confidentiality of patient information was maintained.

### Multiplex immunofluorescence

Paraffin-embedded TNBC tissue sections were deparaffined in xylene and rehydration with ethanol. Antigen retrieval was performed with EDTA antigen repair solution. The slides were blocked in 3% H_2_O_2_ for 30 min at room temperature. The tissues were blocked with 10% goat serum for 30 min at 37°C and then incubated with primary antibodies (**Supplementary Table 2**) at 4°C overnight. The next day, the slides were incubated with secondary antibody at for 45 min at 37°C. Finally, DAPI was used to stain the nuclei and the slides were mounted with fluorescent sealer. These slides were scanned and imaged, and then the mean fluorescence intensities of TK1 and FOXP3 were calculated and statistically analyzed. This analysis was based on the intensity of staining by using Image J analysis software.

### Statistical analysis

Differences in survival between the groups were assessed using Kaplan-Meier curves and the log-rank test. Correlation coefficients were calculated using Pearson and Spearman correlation analyses. Student’s t-test was used to compare continuous normally distributed data, and the Mann-Whitney U test was used to compare non-normally distributed data. For comparisons between more than two groups, the Kruskal-Wallis test and one-way ANOVA were used for nonparametric and parametric data, respectively. All statistical analyses were performed using R software (4.2.2), and *P* values < 0.05 were considered statistically significant.

## Results

### scRNA-seq analysis of normal breast epithelial and TNBC tissues

**Figure 2:**
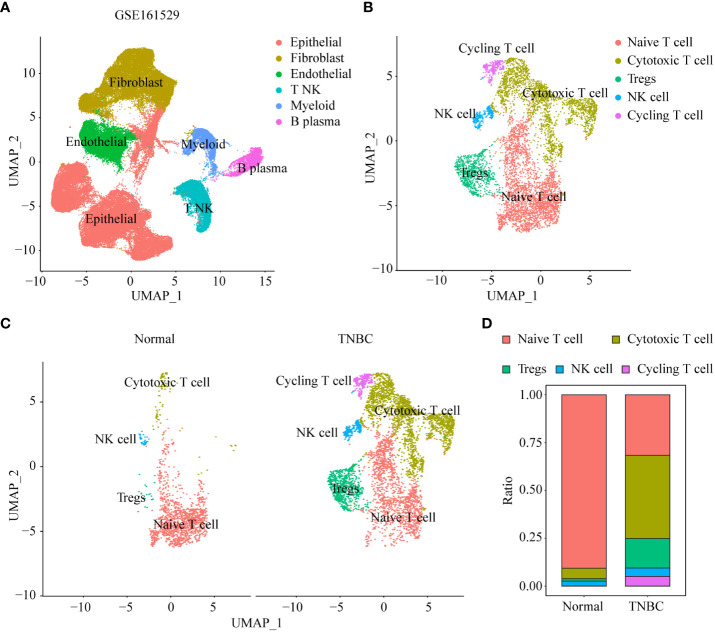
scRNA-seq analysis of normal breast epithelial and TNBC tissues. **(A)** UMAP visualization maps of various cell clusters in normal breast epithelial and TNBC tissues by single cell RNA sequencing analysis. **(B)** UMAP visualization maps of re-clustering annotated NK_T cells in **Figure 2A**. **(C)** The difference in heterogeneity of NK_T cells between normal breast epithelial and TNBC tissues. **(D)** Differences in the proportions of different subtypes of NK_T cells in normal breast epithelial and TNBC tissues.

The scRNA-seq data of 21 samples were extracted and re-analyzed from GSE125449. Of these samples, 13 were from normal breast tissue and eight were from TNBC. We defined six cell clusters using canonical markers: B_plasma, myeloid, NK_T, fibroblast, endothelial, and epithelial cells (**Figure 2A**). To explore the heterogeneity of NK_T cells, we extracted the cells defined as ‘NK_T cells’ and re-clustered the data. Furthermore, these NK_T cells were aggregated into five major populations, including naïve T, regulatory T (Treg), cycling T, cytotoxic T, and NK cells based on known cell markers (**Figure 2B**). Because Treg cells favor the formation of a tumor-suppressive microenvironment, which facilitates tumor progression and reduces the response to immunotherapy, we explored changes in the infiltration of Treg cells during tumor formation. Interestingly, we observed a significantly increased infiltration of Tregs into TNBC tissue compared to normal breast tissue (**Figures 2C, D**).

### Stratification of TNBC tumors based on Treg infiltration

**Figure 3:**
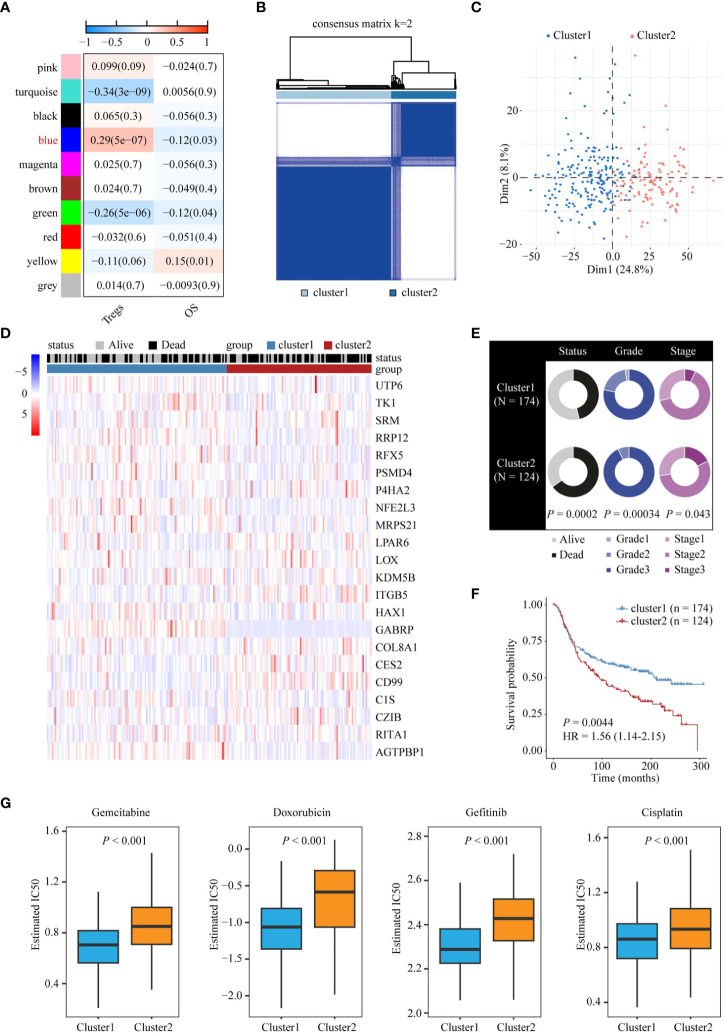
Stratification of TNBC tumors based on Treg infiltration. **(A)** Analysis of correlation coefficients between different phenotypes and co-expression modules. The genes in the blue module positively correlated with Treg infiltration. **(B)** Consensus clustering plot showing METABRIC samples classified into two clusters. **(C)** Principal component analysis of two clusters. **(D)** Heatmap of expression patterns of 22 genes in two clusters. **(E)** Doughnut diagrams of survival status, tumor grade, and tumor stage of two clusters. **(F)** Kaplan-Meier survival analysis shows the difference in OS between the two clusters. **(G)** Differences in chemotherapy responsiveness between the two clusters.

To identify the key marker genes associated with infiltration of Tregs, Treg infiltration was quantified using the CIBERSORT algorithm, and WGCNA was used to detect the gene module related to Treg infiltration. To build a scale-free WGCNA network, we chose a soft-threshold power of β = 5 (**Supplementary Figures 1A, B**). Ten gene modules were identified in the METABRIC cohort, among which the blue gene module was positively correlated with Treg infiltration (r = 0.29, *P* = 5e-07) and negatively correlated (r = 0.12, *P* = 0.03) with patient survival status (**Figure 3A**). We further investigated the correlation between module membership and gene significance for Treg cells, and the results showed that the blue module was highly correlated with Treg cell infiltration (**Supplementary Figure 1C**). Using the survival data available in the METABRIC cohort and the expression values of the blue module genes, we performed survival analysis using a univariate Cox proportional hazards model and identified 22 prognostic genes associated with Treg infiltration. We then performed unsupervised clustering of patients in the METABRIC cohort based on the expression values of these 22 genes. A total of 298 patients in the METABRIC cohort were divided into two groups: 174 patients in Cluster1 and 124 patients in Cluster2 (**Figure 3B**; **Supplementary Figures 2A, B**). PCA also showed that the two groups were distinct (**Figure 3C**). The heatmap (**Figure 3D**) also showed that the expression patterns of these 22 genes differed between Cluster1 and Cluster2. Furthermore, the chi-squared test indicated that patients in Cluster2 exhibited higher tumor grade, stage, and worse survival status than patients in Cluster1 (**Figure 3E**). Survival analysis also showed that the Cluster2 group had a poorer prognosis than the Cluster1 group (**Figure 3F**). Considering that patients with TNBC can develop resistance to multiple chemotherapy drugs, we evaluated the response of these two clusters to four TNBC chemotherapy drugs: gemcitabine, doxorubicin, gefitinib, and cisplatin. We trained the predictive model using ridge regression on the GDSC cell line dataset and evaluated it using 10-fold cross-validation for satisfactory predictive accuracy. We estimated the IC50 for each sample in the METABRIC dataset based on the prediction models for these four chemotherapeutic drugs. The results showed that patients in Cluster1 were more sensitive to these four common chemotherapies (**Figure 3G**). We also investigated the differences in copy number variation (CNV) changes between patients in different clusters using the METABRIC database. Compared with Cluster1, Cluster2, with poorer prognosis, had chromosomal amplification of various tumor-promoting gene loci, such as E2F3, MYC, and GATA3, and partial or complete deletion of the chromosome where the tumor suppressor gene PTEN and RB1 loci are located (**Supplementary Figure 3**).

### Construction and validation of Treg infiltration-related prognostic model

**Figure 4:**
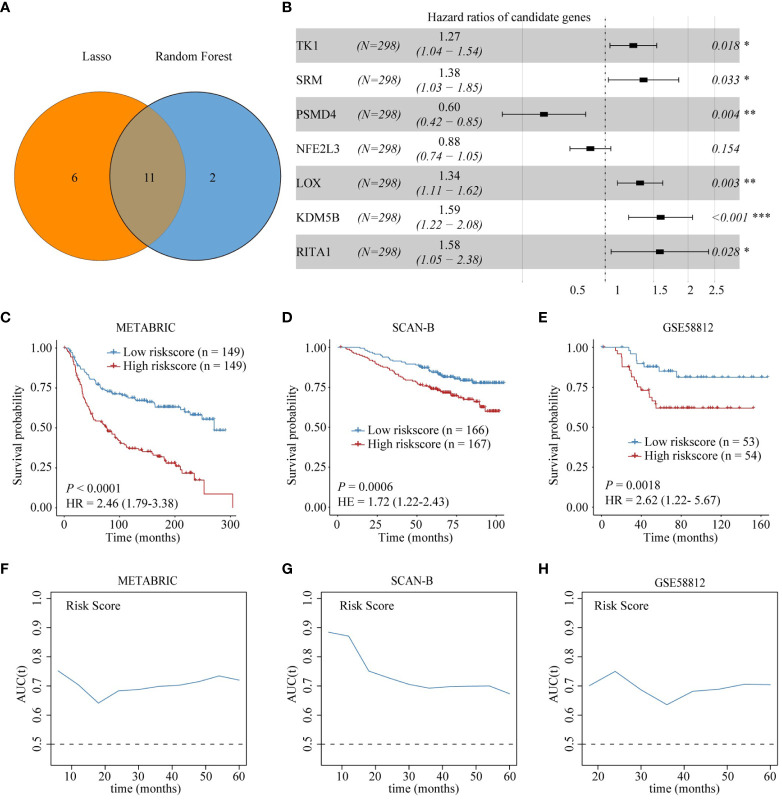
Construction and validation of Treg infiltration-related prognostic model. **(A)** Identification of seven genes in the intersection of the two machine learning algorithms. **(B)** Forest plot shows the results of multivariable Cox proportional hazard regression analysis. **(C-E)** Kaplan-Meier survival analysis shows the difference in OS between high-risk and low-risk score groups in the training and validation sets. **(F-H)** AUC of time-dependent ROC curve for risk score in the training and validation sets.

To facilitate the clinical application of Treg infiltration-related genes in prognosis, we employed two machine learning algorithms to screen key genes from all 22 Treg infiltration-related genes. Seventeen and 13 key genes were identified by LASSO and random forest (**Supplementary Figures 4A, B**), respectively. Then, the seven intersected genes, including TK1, spermidine synthase (SRM), PSMD4, NFE2L3, LOX, KDM5B, and RBPJ interacting and tubulin associated 1 (RITA1), of the two algorithms were selected to construct a multivariate Cox regression model (**Figures 4A, B**). Correlations between each model gene were investigated (**Supplementary Figure 5**). Their respective influence on OS time was explored using Kaplan-Meier survival analysis (**Supplementary Figure 6A**). The Wilcoxon test was applied to explore their expression levels between normal and TNBC tissues in the training cohort (**Supplementary Figure 6B**). The expression of all model genes was notably different, and KDM5B, LOX, SRM, and TK1 were significantly related to prognosis. Patients in the METABRIC cohort were then divided into two groups according to median risk scores, and patients with higher Treg infiltration-related risk scores had poorer OS (**Figure 4C**). In addition, the time ROC curve showed that the risk score had good predictive performance on the OS of patients within 5 years, and the AUC was approximately 0.7 (**Figure 4F**). To validate the prognostic significance of the risk score, we used the same formula to obtain the Treg infiltration-related risk score in two cohorts (GSE58812 and SCAN-B). The risk score had similar prognostic value in these two validation cohorts and good predictive performance for OS (**Figures 4D, E**, **G, H**). Moreover, multivariate Cox analysis suggested that the Treg infiltration-related risk score could be used as an independent prognostic factor for TNBC (**Supplementary Table 3**).

### Differences in immune-related characteristics between high- and low-risk score groups

**Figure 5:**
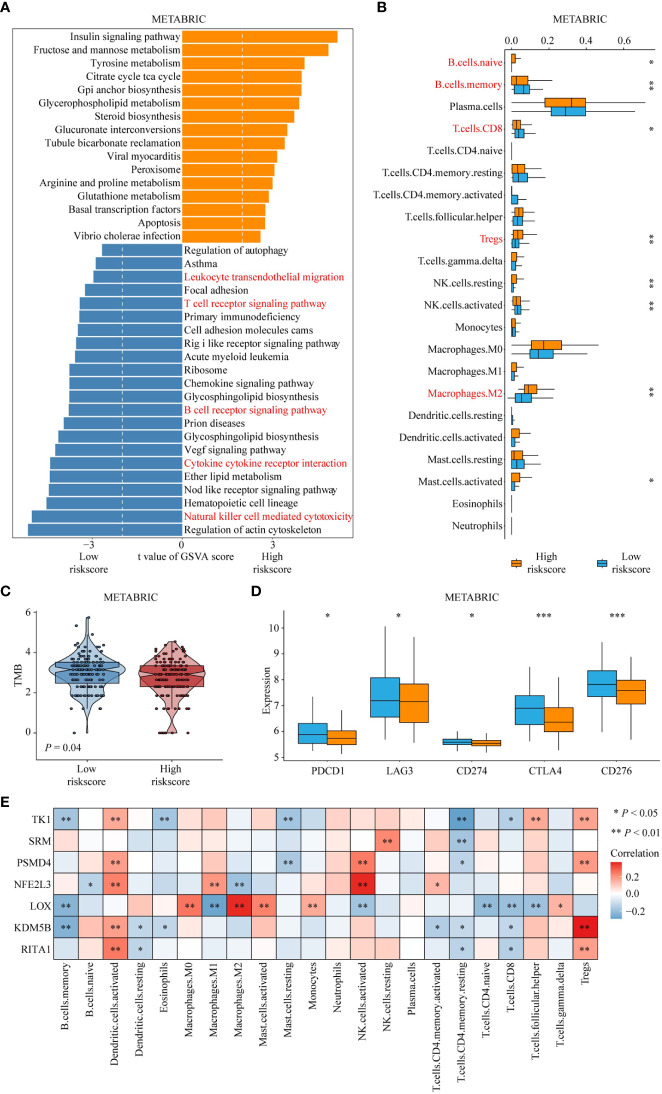
The difference in immune-related characteristics between high- and low-risk score groups. **(A)** Differences in pathway activities scored by GSVA between high- and low-risk score groups. The red fonts emphasize the downregulated immune response-related pathways in patients with high-risk score. **(B)** Differences in immune cell infiltration between high- and low-risk score groups. The red fonts represent enhanced infiltration of immunosuppressive cells and decreased infiltration of tumor-killing immune cells, such as CD8^+^ T cell and B cell in the high-risk group. ‘*’ indicates *P* -value ≤ 0.05, ‘**’ indicates *P*-value ≤ 0.01, ‘***’ indicates *P*-value ≤ 0.001. **(C)** Differences in tumor mutation burden between high- and low-risk score groups. **(D)** Differences in expression of known immune checkpoint genes between high- and low-risk score groups. **(E)** Correlation of risk score genes with immune cell infiltration.

To reveal the difference in pathway activation for each risk score group, we calculated the GSVA score based on KEGG pathway gene sets. As shown in **Figure 5A**, patients with high-risk scores had higher activation of metabolism-related pathways, such as lipid and amino acid metabolism-related pathways, which was consistent with the reported metabolic properties of Treg cells. However, patients with a high-risk score showed lower activation of immune-related pathways, such as the T- and B-cell receptor signaling pathways (**Figure 5A**). Furthermore, the results of the CIBERSORT algorithm for the METABRIC cohort and SCAN-B showed lower B-cell and CD8 T-cell infiltration but higher Treg-cell and M2-macrophage infiltration in high-risk score patients relative to low-risk score patients (**Figure 5B**; **Supplementary Figure 7A**). Interestingly, the tumor mutational burden (TMB) level in the high-risk group was lower than that in the low-risk group (**Figure 5C**). Moreover, we identified the difference in immune checkpoint expression between low- and high-risk score groups, showing lower expression of immune checkpoint genes in patients with high-risk scores than in those with low-risk scores (**Figure 5D**, **Supplementary Figure 7B**). Finally, we calculated the correlation between the risk score genes and immune cell infiltration. The results showed that most of the genes were negatively correlated with immune cell infiltration that promotes immunotherapy response (B cells, CD8 T cells) and positively correlated with Treg cell infiltration (**Figure 5E**).

### Estimation of drug and immunotherapy responses

**Figure 6:**
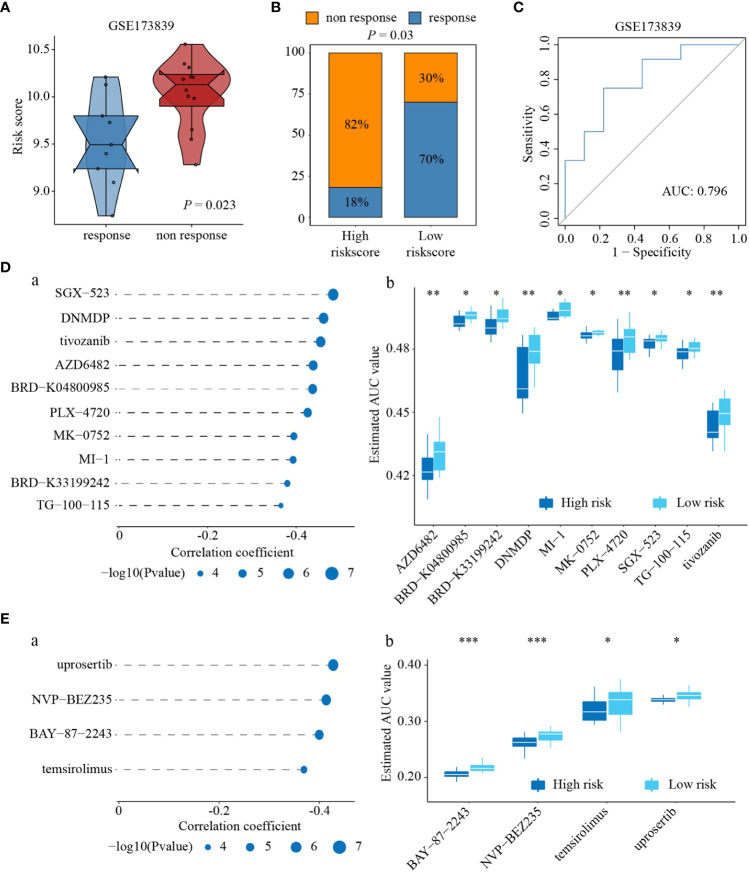
Estimation of drug and immunotherapy responses. **(A)** Differences in risk score between response to immunotherapy and non-response to immunotherapy. **(B)** Differences in proportion of patients who failed to response to immunotherapy between high- and low-risk score groups. **(C)** AUC of risk score on predicting immunotherapy effectiveness. **(D)** Spearman correlation and differential response analyses of ten CRTP-derived compounds (a), and the difference of AUC value between high- and low-risk score groups response to ten CRTP-derived compounds (b). **(E)** Spearman correlation and differential response analyses of four PRISM-derived compounds (a), and the difference of AUC value between high- and low-risk score groups response to four PRISM-derived compounds (b). ‘*’ indicates P-value ≤ 0.05, ‘**’ indicates P-value ≤ 0.01, ‘***’ indicates P-value ≤ 0.001.

The above results suggest that the Treg infiltration-related risk score is associated with immunotherapy effectiveness. We collected a dataset containing immunotherapy data for patients with TNBC, GSE173839. Box plots showed higher risk scores in immunotherapy non-responders than in immunotherapy responders (*P* = 0.023, **Figure 6A**). The percentage of patients who failed to respond to immunotherapy in the high-risk score group was higher than that in the low-risk score group (82% VS 30%, **Figure 6B**). In addition, ROC curve analysis indicated that the Treg infiltration-related risk score had excellent performance in predicting immunotherapy responsiveness (AUC = 0.796, **Figure 6C**). We employed two different approaches to identify drug candidates with higher drug sensitivity in patients with a high Treg infiltration-related risk score. Analyses were performed using CTRP- and PRISM-derived drug response data. First, a differential drug response analysis was performed between the high Treg infiltration-related risk score and low Treg infiltration-related risk score groups to identify compounds with lower estimated AUC values in the high Treg infiltration-related risk score group (log2FC > 0.10). Next, we used the Spearman correlation coefficient between AUC values and Treg infiltration-related risk score to select compounds with negative correlation coefficients (Spearman’s r < -0.50 for CTRP or -0.45 for PRISM). These analyses yielded ten CTRP-derived compounds (including SGX-523, DNMDP, tivozanib, AZD6482, BRD-K04800985, PLX-4720, MK-0752, MI-1, BRD-K33199242, and TG-100-115) (**Figure 6D**a) and four PRISM-derived compounds (including uprosertib, NVP-BEZ235, BAY-87-2243, and temsirolimus) (**Figure 6E**a). All these compounds had lower estimated AUC values in the high Treg infiltration-related risk score group and were inversely correlated with the Treg infiltration-related risk score (**Figure 6D**d, **E**b).

### Nomogram establishment and assessment

**Figure 7:**
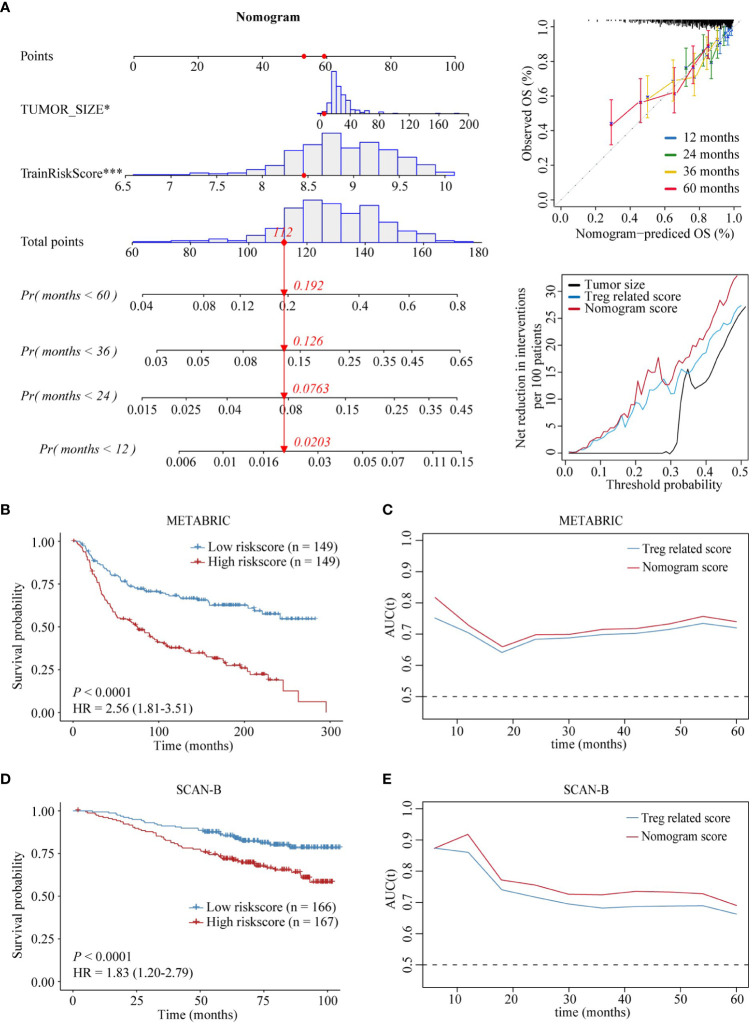
Establishment and assessment of the nomogram model. **(A)** Nomogram based on Treg infiltration-related risk score and tumor size (left). Disease-specific survival calibration curves at 1, 2, 3, and 5 years (right, up). Nomogram decision curve analysis, Treg infiltration-related risk score, and tumor size (right, down). **(B-E)** Prognostic value of the nomogram in the training and validation sets. ‘*’ indicates P-value ≤ 0.05, ‘***’ indicates P-value ≤ 0.001.

To enhance the predictive power of the above risk scores, risk score and tumor size were combined to establish a nomogram model using multivariable Cox regression analysis (**Figure 7A**). Disease-specific survival (DSS) calibration curves at 1, 2, 3, and 5 years showed that predicted survival probabilities were in accordance with actual survival, demonstrating the robustness of this nomogram in predicting survival (**Figure 7A**). In addition, DCA was performed, and the results showed that the prognostic value of the nomogram was superior to that of the individual variables (**Figure 7A**). Furthermore, the results of our study showed that the AUC predicted by the nomogram exceeded that of the risk score ​​in both the training set and the test cohort (**Figures 7B-E**).

### TK1 serves as a key risk score player and tumor promoter in TNBC

**Figure 8:**
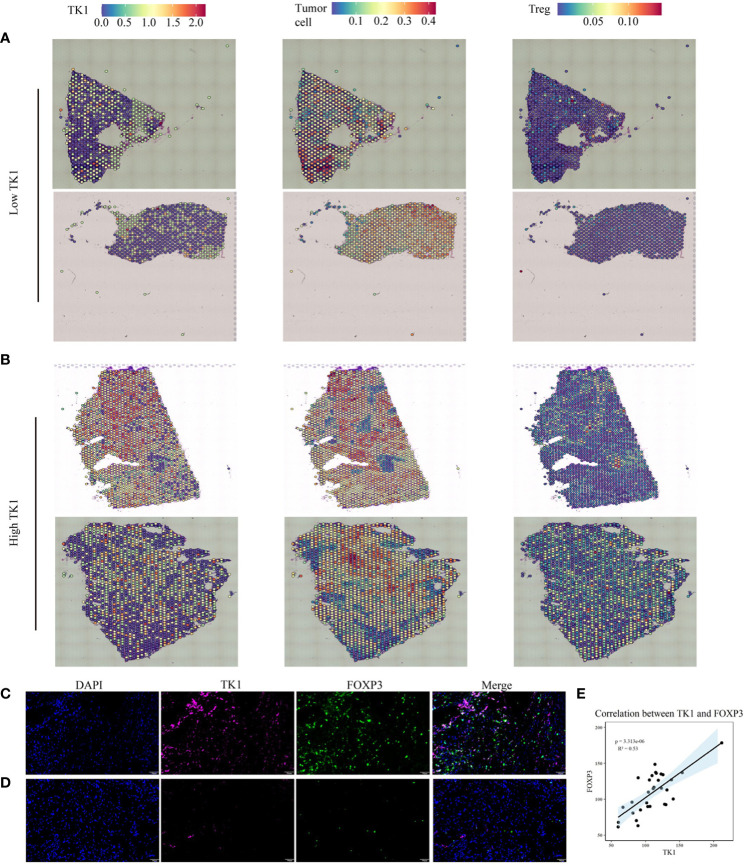
Multiplex immunofluorescence and spatial transcriptomics were employed to investigate the correlation between tumor cells expressing TK1 and the infiltration of Treg cells. **(A, B)** The relationship between TK1-expressing tumor cells and Treg cell infiltration. **(C-E)** High TK1 expression associated with increased Treg infiltration. Representative images of multiplex immunofluorescence in TK1-high **(C)** and TK1-low **(D)** human triple-negative breast cancer tissues. **(E)** Statistical analysis of the mean fluorescence intensity of Foxp3 and TK1. Blue: DAPI; Magenta: TK1; Green: Foxp3. Scale bar = 50 µm.

Results of the spatial transcriptome analysis reveals that TK1-positive expressed cells primarily localize in the tumor cells. Furthermore, it demonstrates an elevated infiltration of Treg cells in TNBC tissues with high expression of TK1 (**Figures 8A, B**). The Treg marker-FOXP3 multiple fluorescent staining method was used to evaluate 31 cases of patients with TNBC to detect the relationship between TK1 expression and Tregs. As expected, TK1 expression was positively correlated with the expression status of the Treg marker Foxp3 (**Figures 8C-E**). This result suggested that patients with higher TK1 expression exhibited more Treg infiltration.

**Figure 9:**
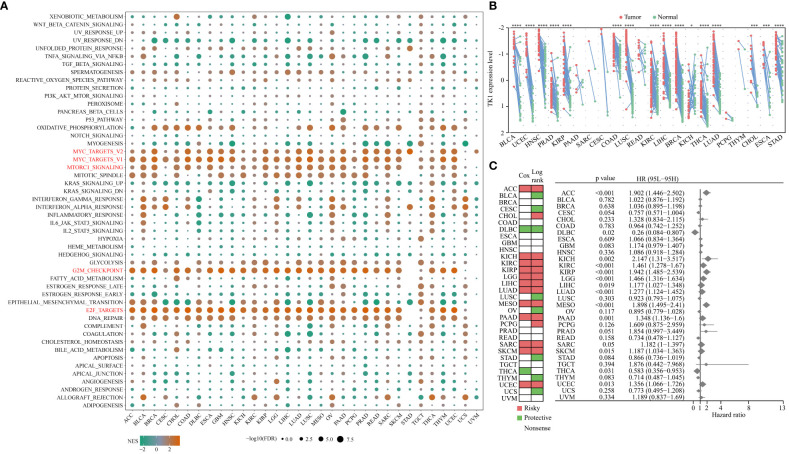
Relationship between TK1 and Hallmark pathways in TCGA pan-cancer cohorts. **(A)** NES is the normalized enrichment score in the GSEA algorithm. **(B)** Differential expression of TK1 in tumor tissue relative to normal tissue among 20 cancer types in the pan-cancer TCGA cohort. X-fold changes as compared to normal tissue are shown. **(C)** Summary of the relationship between TK1 expression and OS across 33 cancer types in the TCGA pan-cancer cohort. ‘*’ indicates P-value ≤ 0.05, ‘***’ indicates P-value ≤ 0.001, ‘****’ indicates P-value ≤ 0.0001.

The relationship between TK1 and the Hallmark pathway in the TCGA pan-cancer cohort was then analyzed. The results of this study showed that TK1 was positively correlated with cell cycle- and proliferation-related pathways, such as MYC target and MTORCI signaling pathway, in multiple cancer types (**Figure 9A**). TK1 expression in tumor and normal tissues of 20 cancer types in the TCGA cohort was analyzed; TK1 was upregulated in 70% of tumors, including BLCA, UCEC, HNSC, PRAD, KIRP, COAD, LUSC, KIRC, LIHC, BRCA, THCA, LUAD, CHOL, ESCA, and STAD (**Figure 9B**). We analyzed the relationship between TK1 and the survival prognosis of patients with 33 types of cancer, and the results showed that high TK1 expression was associated with impaired survival in more than 10 cancers (**Figure 9C**).

**Figure 10:**
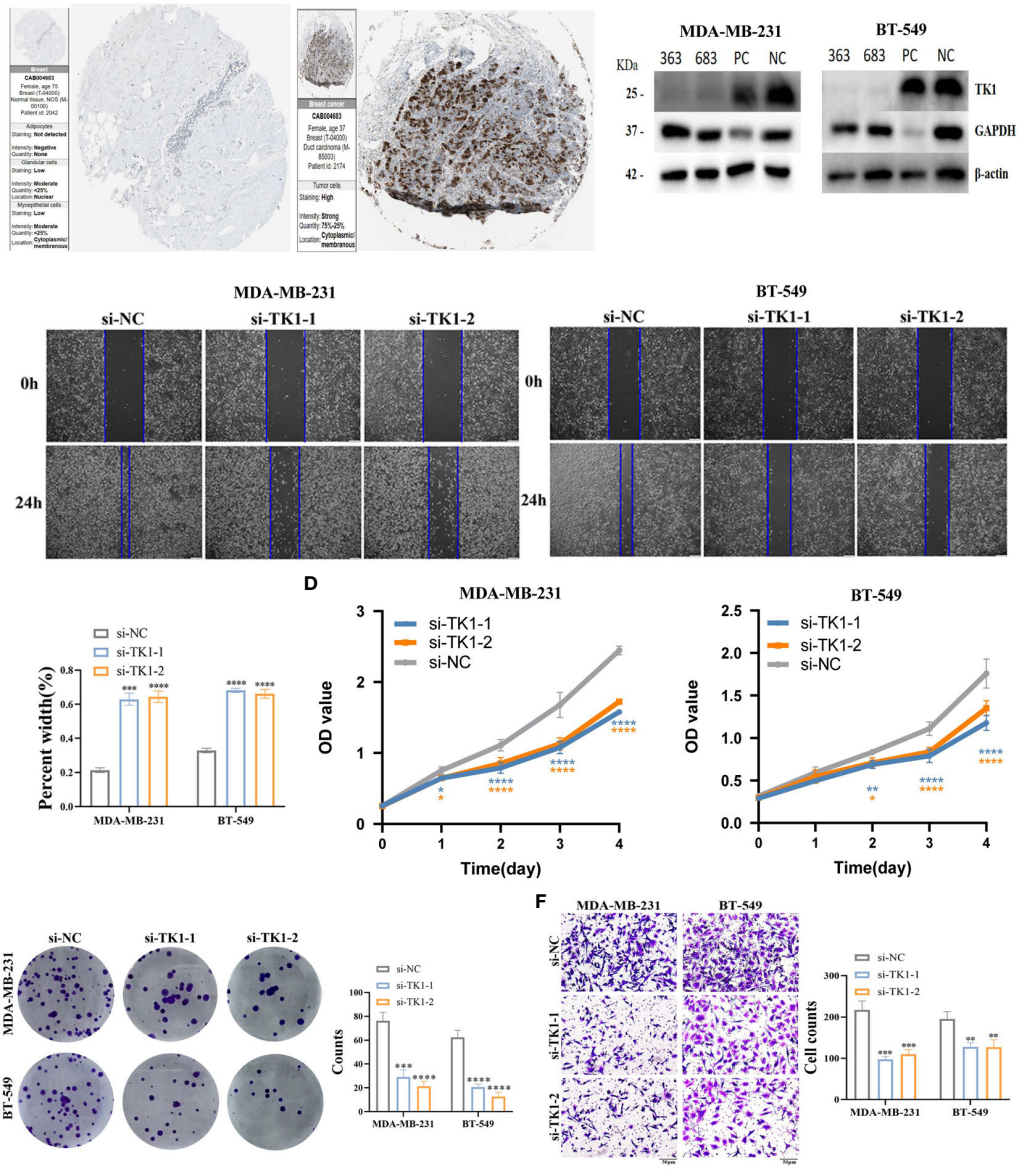
Verification of TK1 for proliferation and migration in TNBC. **(A)** TK1 protein levels in normal breast and breast cancer were visualized by IHC in HPA. **(B)** Construction and verification of siRNA specifically targeting TK1 (si-TK1-1, si-TK1-2). The biological functions of TK1 on TNBC cell lines were verified via wound healing **(C)**, CCK-8 **(D)**, colony formation **(E)** and transwell **(F)** experiments. ‘*’ indicates P-value ≤ 0.05, ‘**’ indicates P-value ≤ 0.01, ‘***’ indicates P-value ≤ 0.001, ‘****’ indicates P-value ≤ 0.0001.

To verify the role of TK1 as a key player in the risk score as well as a tumor promoter in TNBC, we performed several functional experiments to detect the migration, invasion, and proliferation abilities of the control and siRNA-mediated knockdown groups. The HPA database shows that TK1 is highly expressed in cancer tissues (**Figure 10A**). To explore the role of TK1 in TNBC proliferation and migration *in vitro*, transient transfection targeting TK1 was used to inhibit its expression (si-TK1-1 and si-TK1-2). siRNA-mediated knockdown reduced TK1 expression in MDA-MB-231 and BT-549 cells (**Figure 10B**). The migration, invasion, and proliferative abilities of TNBC cells decreased after TK1 knockdown, and the differences were statistically significant. Compared with those in the control group, the results of CCK-8 (**Figure 10D**) and colony formation assays (**Figure 10E**) showed that silencing TK1 significantly reduced the viability and proliferative ability of TNBC cells, respectively. Moreover, results of the wound-healing assay and transwell assay also revealed that the migration ability of cells after TK1 knockdown was decreased compared with the control group (**Figures 10C, F**). In conclusion, these findings collectively confirm that TK1 promotes the proliferation and migration of TNBC cells.

## Discussion

TNBC is known for its aggressive behavior, including early recurrence and metastasis, and remains a lethal disease owing to its high heterogeneity and limited treatment options (24). Currently, chemotherapy remains the standard treatment for TNBC (25–27). Unfortunately, patients often develop resistance to chemotherapy (28), highlighting the urgent need to identify new therapeutic targets for TNBC.

In recent years, advances in omics technologies have revealed the relevance of tumor microenvironment heterogeneity in TNBC and uncovered its close dynamic relationship with cancer cell characteristics (29). In our study, using scRNA-seq technology, a powerful tool for investigating tumor heterogeneity and cellular subpopulations (29), we observed a substantial increase in Treg infiltration within TNBC tissue compared to normal breast tissue. Furthermore, we revealed two Treg infiltration-related subtypes in TNBC; Cluster1 tended to represent a more severe condition than Cluster2. This finding is consistent with previous studies indicating that Treg infiltration worsens TNBC progression, leads to poorer prognosis, and contributes to immunotherapy resistance (30–38). For example, Bai et al. demonstrated that high annexin-A1 expression in Tregs was associated with poor prognosis in TNBC, and inhibiting the function of tumor-infiltrating Tregs could reduce the size of TNBC tumors (39). Li et al. demonstrated that chemokine (C-X-C motif) ligand 1 derived from tumor-associated macrophages induces the establishment of an immune-suppressive TME by recruiting naïve CD4+ T cells and promoting their differentiation into Tregs (40). It is necessary to assess the risk associated with Treg infiltration in TNBC and future research should explore targeted modulation of Tregs to develop new treatment options for patients with TNBC.

Furthermore, the seven intersected genes were selected to construct the multivariate Cox regression model. The algorithmic analysis of the three cohorts revealed that Treg infiltration-related risk scores categorized patients into high- and low-risk groups, with the high-risk group exhibiting worse OS decreased infiltration of B and CD8 T cells, and increased infiltration of Tregs compared to the low-risk group. Numerous studies have demonstrated the significance of T- and B-cell immune signaling pathways as important features for predicting prognosis, which are associated with favorable responses to immunotherapy and better clinical outcomes. Tregs have been shown to suppress the cytotoxic function of CD8+ T cells, support B-cell growth, and promote cancer progression (41–45). This suggests that Treg infiltration, along with the resulting lack of immune response pathways, is a main reason for the inferior outcomes observed in the high-risk group compared to the low-risk group. To explore the potential mechanisms underlying the differences in survival between different risk groups, we further investigated a series of pathway enrichment analyses and found that patients with high-risk scores showed higher activation levels of metabolism-related pathways, such as lipid and amino acid metabolism, which aligns with the reported metabolic characteristics of Treg cells (46, 47).

Studies have shown that the prognostic model genes TK1, LOX, KDM5B, PSMD4, and NFE2L3 are associated with breast cancer progression, prognosis stratification, and clinical drug resistance (48–58). Among these, TK1 exhibited the most significant prognostic value in our study. TK1 is a cell cycle-dependent kinase that catalyzes the addition of γ-phosphate to thymidine and participates in the pyrimidine nucleotide salvage pathway (17). TK1 is universally recognized as a hallmark of cellular proliferation and elicits oncogenic effects in diverse malignant neoplasms. Our investigation elucidated the interrelationship between TK1 and cell cycle-associated cascades, as well as proliferation-linked pathways across a spectrum of cancer types (59–62). As expected, we confirmed that TK1 was positively correlated with cell cycle- and proliferation-related pathways, such as MYC target and MTORCI signaling pathway, in multiple cancer types. In addition, we also verified TK1 *in vitro*, demonstrating that TK1 down-regulation can inhibit the proliferation, migration, and invasion of TNBC cells. Meanwhile, TK1 activity was found to be significantly higher in patients with breast cancer (BC) than in healthy women (63–67). Nisman et al. identified TK1 activity as an independent prognostic factor for recurrence-free survival and found elevated TK1 levels in BC tumors with increased proliferative activity (66). TK1 is associated with aggressive tumor features such as advanced stage, high grade, ER/PgR negative, tumor necrosis, and vascular invasion, suggesting its central role as a BC proliferation marker (66). Fanelli et al. proposed that using a 2.5% TK1 cutoff value could be a useful tool for prognostic or predictive purposes in BC tissues (51). Therefore, TK1 not only holds promise as a potential biomarker for TNBC recurrence, treatment monitoring, and survival, but may also offer advantages over current biomarkers.

FOXP3 is a transcription factor that inimitably defines Tregs and is a requirement for Treg differentiation (68). FOXP3-expressing Treg cells, which are characterized by FOXP3, play a critical role in maintaining immune homeostasis by suppressing self-reactive T cells and other cells (69, 70). The ratio of FOXP3+ T cells to CD3 or CD8+ T cells is inversely correlated with the survival rate of multiple cancers (71–73). Studies have shown that a higher number of FOXP3-positive Tregs identifies patients with BC with no recurrence and shorter OS, suggesting that Tregs may impede anti-tumor immune responses (33, 74, 75). Moreover, our study revealed that TK1 expression was positively correlated with the expression status of the Treg marker FOXP3, which means that high TK1 expression in TNBC cells is positively correlated with the risk of Treg infiltration. This suggests that stimulation of TK1 signaling in TNBC cells may promote Treg differentiation and induction, indicating that Treg infiltration-related risk scoring can serve as an independent prognostic factor in TNBC. Comprehensive analysis demonstrated that Treg infiltration-associated genes can be utilized to establish clinically relevant TNBC progression classifications, and the evaluated Treg score can function as an independent prognostic biomarker for TNBC, further advancing precise TNBC immunotherapy.

Although a few targeted therapies, including poly (ADP-ribose) polymerase inhibitors (e.g., olaparib and talazoparib) and immune checkpoint inhibitors (e.g., atezolizumab and pembrolizumab), have been approved for TNBC, their clinical benefits are limited to a small subset of patients with BRCA1/2 mutations or programmed death ligand 1 expression (27, 68, 76). Developing novel targeted therapies with different mechanisms of action for TNBC can work synergistically with these approved modalities, potentially further enhancing clinical efficacy (69).

TMB, defined as the number of somatic mutations per megabase of the genome, is more likely to respond to immune checkpoint inhibitors (70–72). In our study, we found that TMB levels were lower in the high-risk group than in the low-risk group, indicating a weaker response to immunotherapy in high-risk patients. In a dataset (GSE173839) of patients with TNBC undergoing immunotherapy, non-responders exhibited higher risk scores than responders, and the high-risk score group had a higher proportion of patients with ineffective immunotherapy, highlighting the predictive efficacy of the Treg infiltration-related risk score for immunotherapy responsiveness. Extensive research has shown that chemical inhibition is the most appropriate choice for downregulating Treg-related immune suppression (73–75, 77). Therefore, we conducted analyses using drug response data derived from CTRP and PRISM, ultimately identifying 10 CTRP-derived compounds (SGX-523, DNMDP, tivozanib, AZD6482, BRD-K04800985, PLX-4720, MK-0752, MI-1, BRD-K33199242, and TG-100-115) and four PRISM-derived compounds (uprosertib, NVP-BEZ235, bay87-2243, and temsirolimus). In the subset with high Treg infiltration-related risk scores, all compounds exhibited lower estimated AUC values and showed a negative correlation with Treg infiltration-related risk score, thereby identifying candidate drugs with higher sensitivity. Previous studies have indicated a crucial role of tumor-infiltrating Tregs in TNBC immune tolerance, antitumor immunity, and immune evasion (46, 47, 78, 79). Gajewski et al. demonstrated that tumor-infiltrating Tregs promote immune evasion via the expression of T-cell markers, type I interferon markers, programmed cell death-ligand 1, indoleamine 2, 3-dioxygenase, and FOXP3 (80). Semba et al. confirmed that JNK-regulated TAM-produced C-C motif chemokine 2 promotes TNBC invasiveness by recruiting Tregs, thereby facilitating an immunosuppressive TME (47). Oshi et al. found a significant correlation between low Treg abundance and pathological complete response after neoadjuvant chemotherapy in TNBC (81). Therefore, targeting Tregs may improve the treatment prognosis of patients with TNBC. Furthermore, multiple patterns of programmed cell death have been proposed as an ideal predictive model for assessing the progression and drug sensitivity in postoperative TNBC patients. This model can accurately predict the patients’ prognosis and drug sensitivity after TNBC surgery (82).

To enhance the predictive ability of the risk score, we combined it with tumor size and employed multivariate Cox regression analysis to establish a nomogram model. Additionally, we constructed a column chart to visualize and predict the 1-, 2-, 3-, and 5-year survival probabilities of patients, demonstrating the higher predictive accuracy of the column chart. Therefore, this column chart can guide the establishment of personalized screening programs for patients with TNBC and facilitate the efficient utilization of medical resources. However, this study has a few potential limitations. First, all the cohort studies were retrospective, necessitating future validation through multicenter, large-sample, prospective double-blind trials. Second, the analysis encompassed data from all patients with TNBC in the METABRIC cohort without considering molecular subtypes due to limited samples and individual tumor cells. Third, further confirmation of drug sensitivity through cellular experiments and additional animal studies exploring the functional role of TK1 in TNBC would provide stronger clues for guiding clinical applications.

## Conclusions

In this study, we demonstrated that Treg infiltration-related genes in TNBC could be used to establish clinically relevant TNBC classifications. Based on three TNBC cohorts, we developed and validated a Treg infiltration-related prognostic model, identifying the role of Treg infiltration-related genes in the development of the tumor immune microenvironment and immune therapy response. Furthermore, we revealed that Tregs may affect tumor occurrence, progression, and prognosis by modulating the expression of TK1 within the TME. We identified candidate drugs with a negative correlation to Treg infiltration-related risk scores and higher drug sensitivity, which can predict TNBC clinical outcomes and immunotherapy response. We believe that our Treg infiltration-related prognostic model can broaden our understanding of TNBC biology and prognostic prediction, and targeting Tregs presents a promising therapeutic approach for TNBC.

## Data availability statement

The original codes used for analyses presented in the study are publicly available. This data can be found here: https://github.com/TNBC222/TNBC_Tregs and 10.6084/m9.figshare.23750538.

## Ethics statement

The studies involving humans were approved by Hospital Review Board of Tianjin Union Medical Center. The studies were conducted in accordance with the local legislation and institutional requirements. The human samples used in this study were acquired from primarily isolated as part of your previous study for which ethical approval was obtained. Written informed consent for participation was not required from the participants or the participants’ legal guardians/next of kin in accordance with the national legislation and institutional requirements. Written informed consent was obtained from the individual(s) for the publication of any potentially identifiable images or data included in this article.

## Author contributions

PH: Data curation, Methodology, Writing – original draft. XZ: Data curation, Methodology, Writing – original draft. MZ: Data curation, Formal Analysis, Writing – original draft. YY: Data curation, Formal Analysis, Writing – original draft. GJ: Conceptualization, Writing – original draft. SZ: Conceptualization, Supervision, Writing – review & editing.
