# Supplementary material for: Regulatory T cells are associated with the tumor immune microenvironment and immunotherapy response in triple-negative breast cancer
Source: Front Immunol. 2023 Sep 12;14:1263537. doi: 10.3389/fimmu.2023.1263537 (PMC10521732; doi:10.3389/fimmu.2023.1263537)
Supplement: Supplementary file 1 [file DataSheet_1.docx]

**Supplementary figure legends**

**
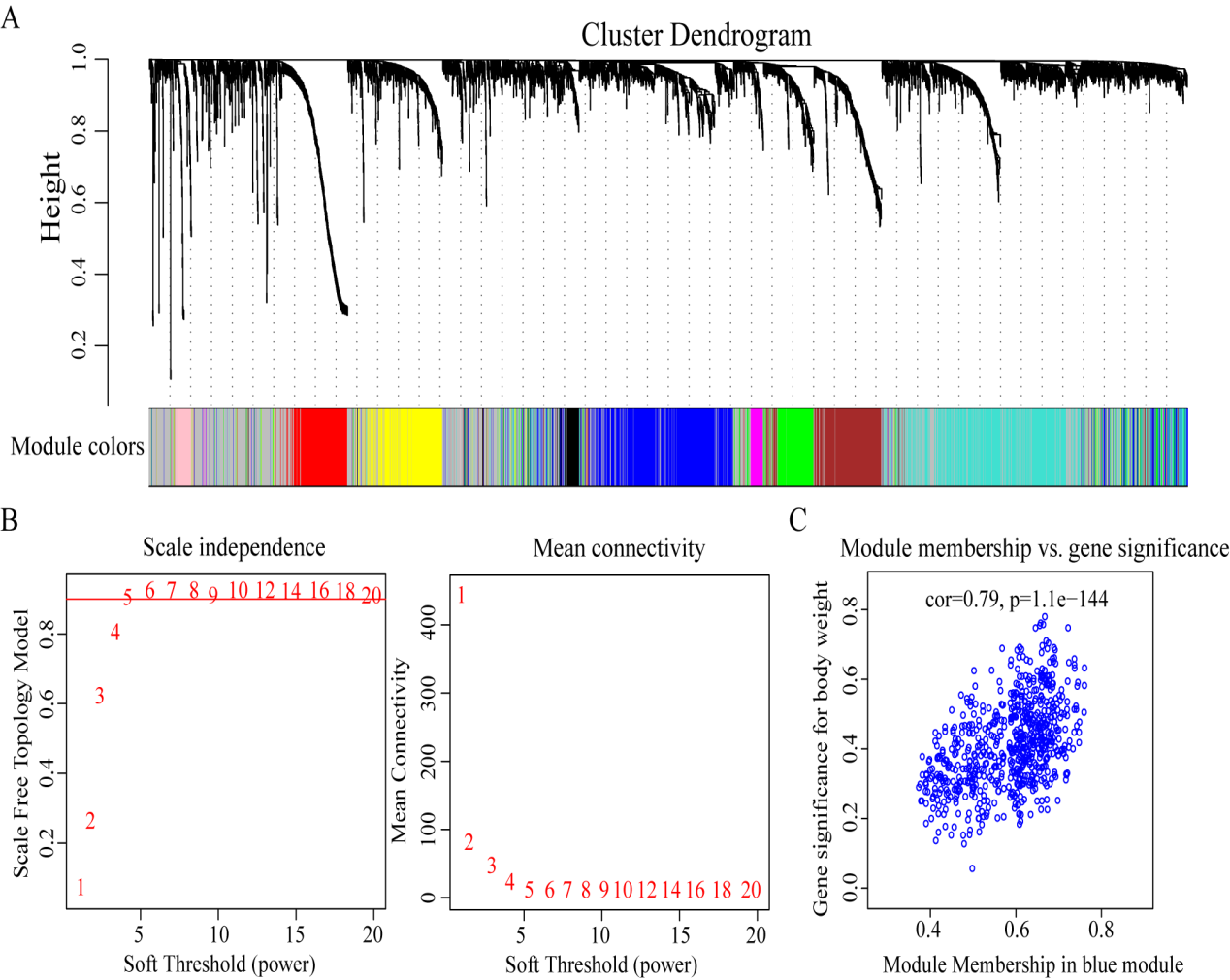
**

**Supplementary figure S1. The results of WGCNA in the METABRIC cohort.** (A) The hierarchical gene dendrogram and module color of the METABRIC cohort. (B) Scale-free fit index and mean connectivity for different soft-threshold powers (β). (C) Correlation between module membership and gene significance for Treg cell infiltration.


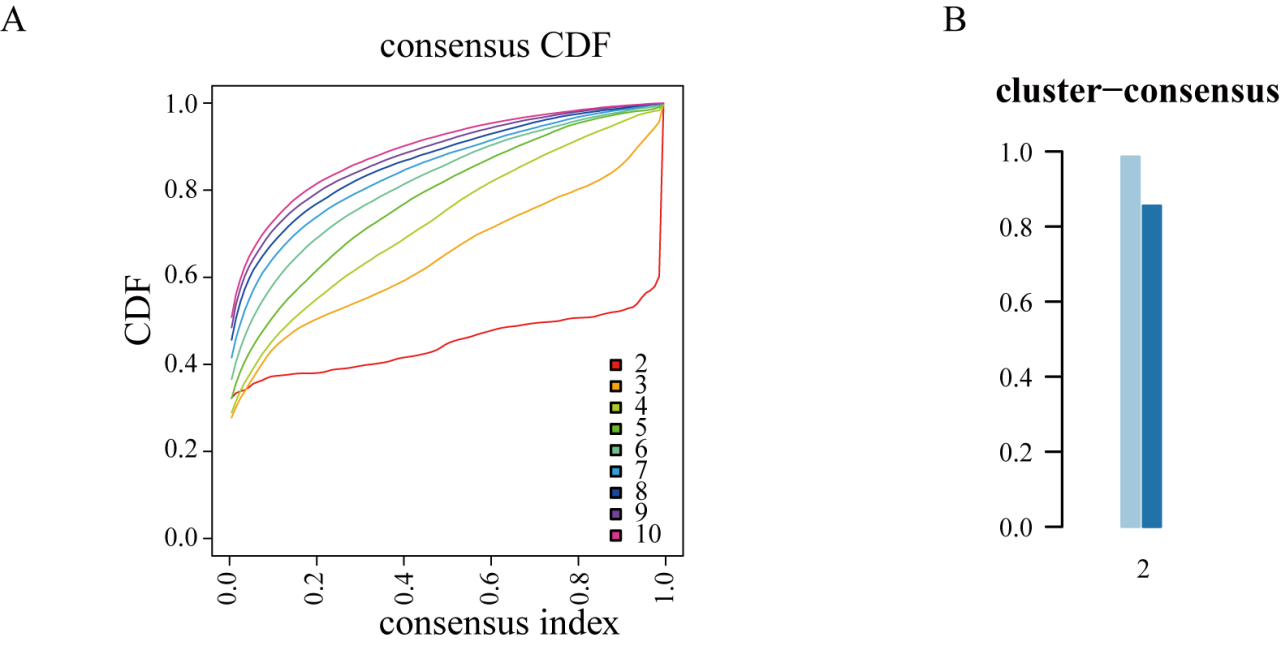


**Supplementary figure S2. Defining the stable cluster of consensus clustering.** (A-B) The cumulative density function (CDF) ranges from two to ten consensus groups. A delta curve is a graph of the CDF series showing the relative change in the area under the CDF curve.

**
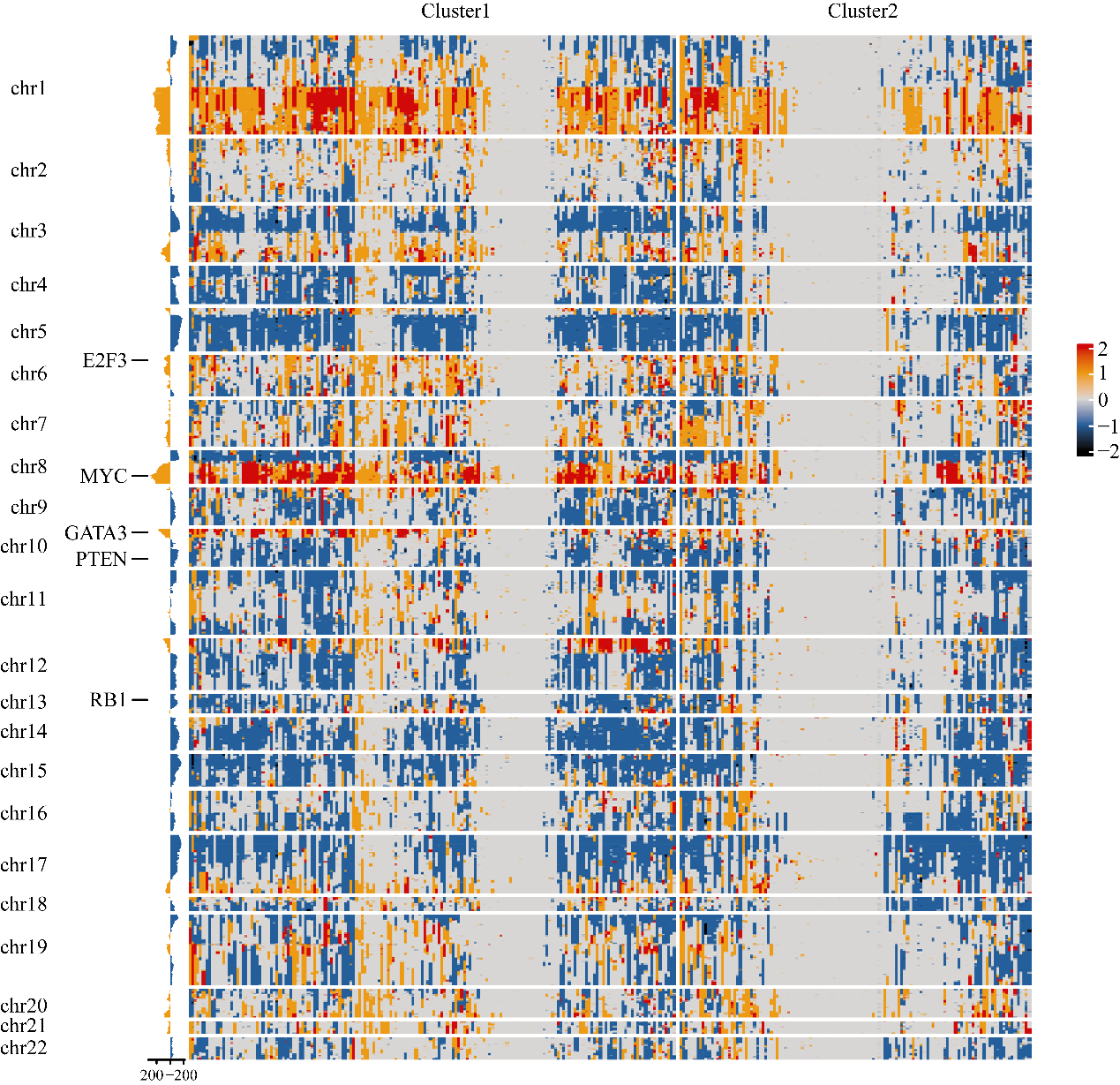
**

**Supplementary figure S3. Differences in copy number alterations across clusters.** Gains and losses are summarized on the left side of the Chromosome bar panel.

**
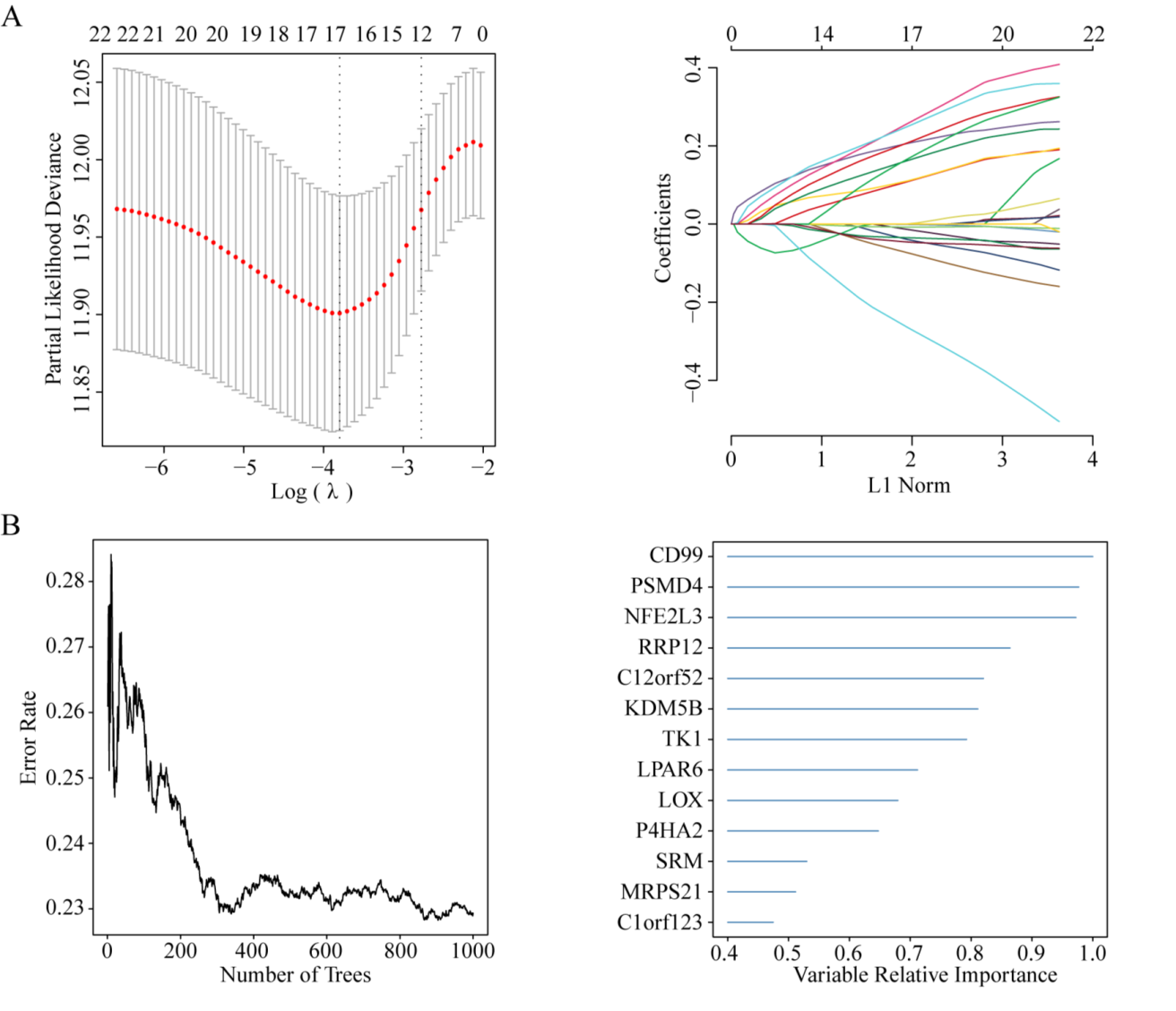
**

**Supplementary figure S4. Feature selection of two machine learning algorithms.** (A) LASSO regression analysis screens feature genes with patient overall survival. (B) Random survival forest explores genes of importance associated with patient overall survival.


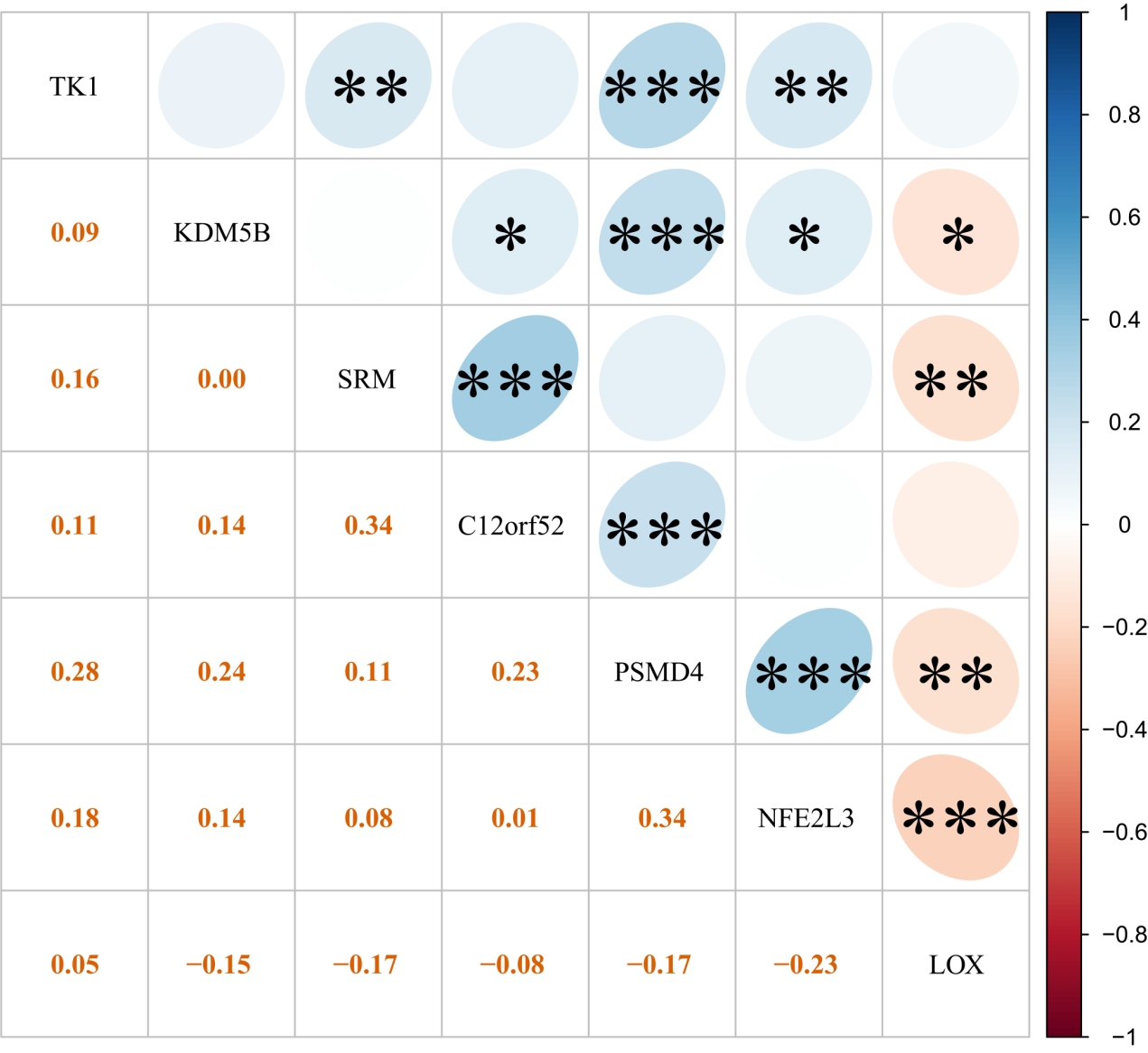


**Supplementary figure S5.** TK1 positively correlated with SRM, PSMD4, and NFE2L3 in METABRIC TNBC samples.

**
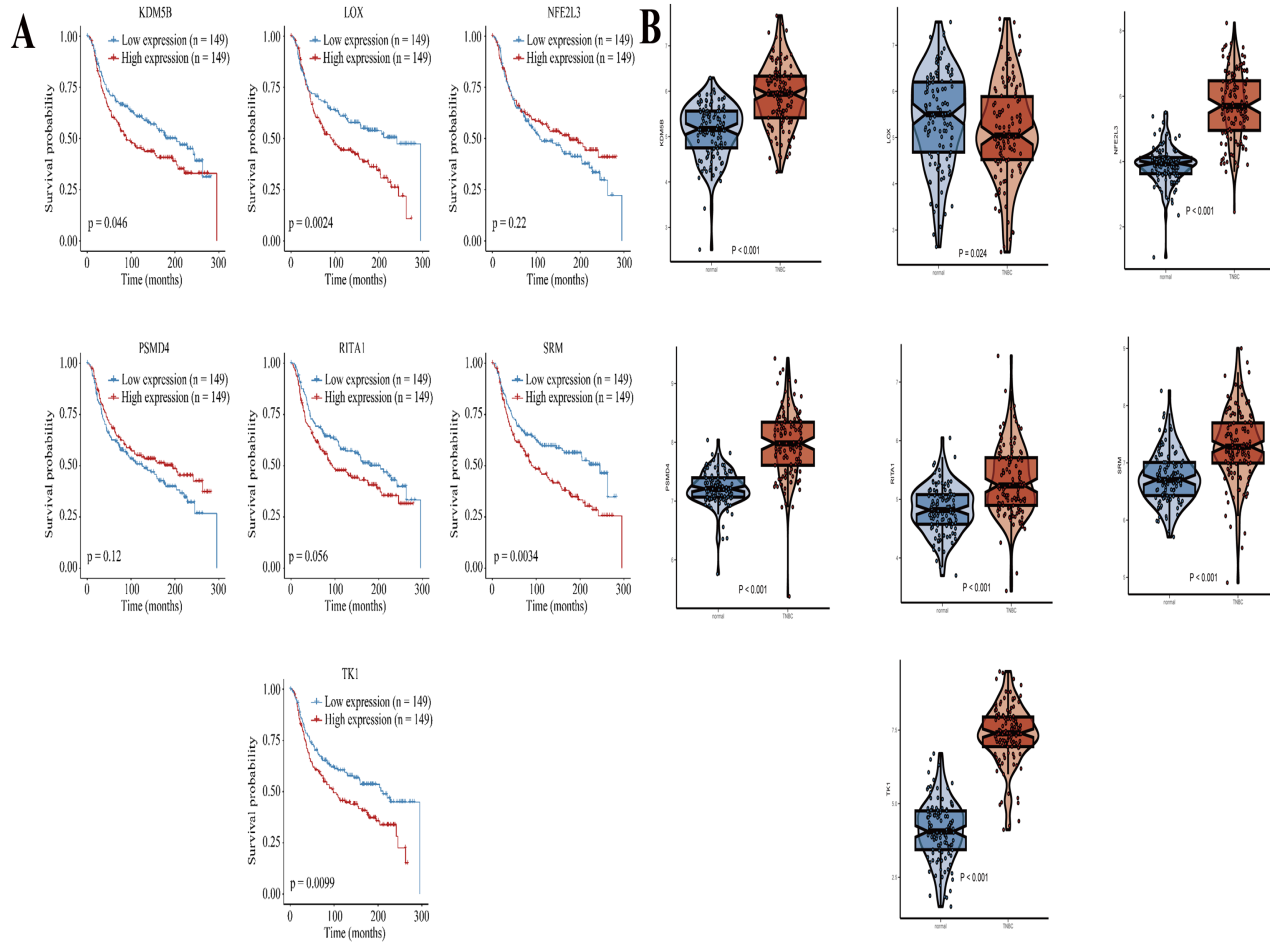
**

**Supplementary figure S6. Prognostic value and expression levels of each model gene.** (A) Kaplan-Meier analysis of each model gene. (B) The expression levels of each model gene between TNBC and normal tissues.


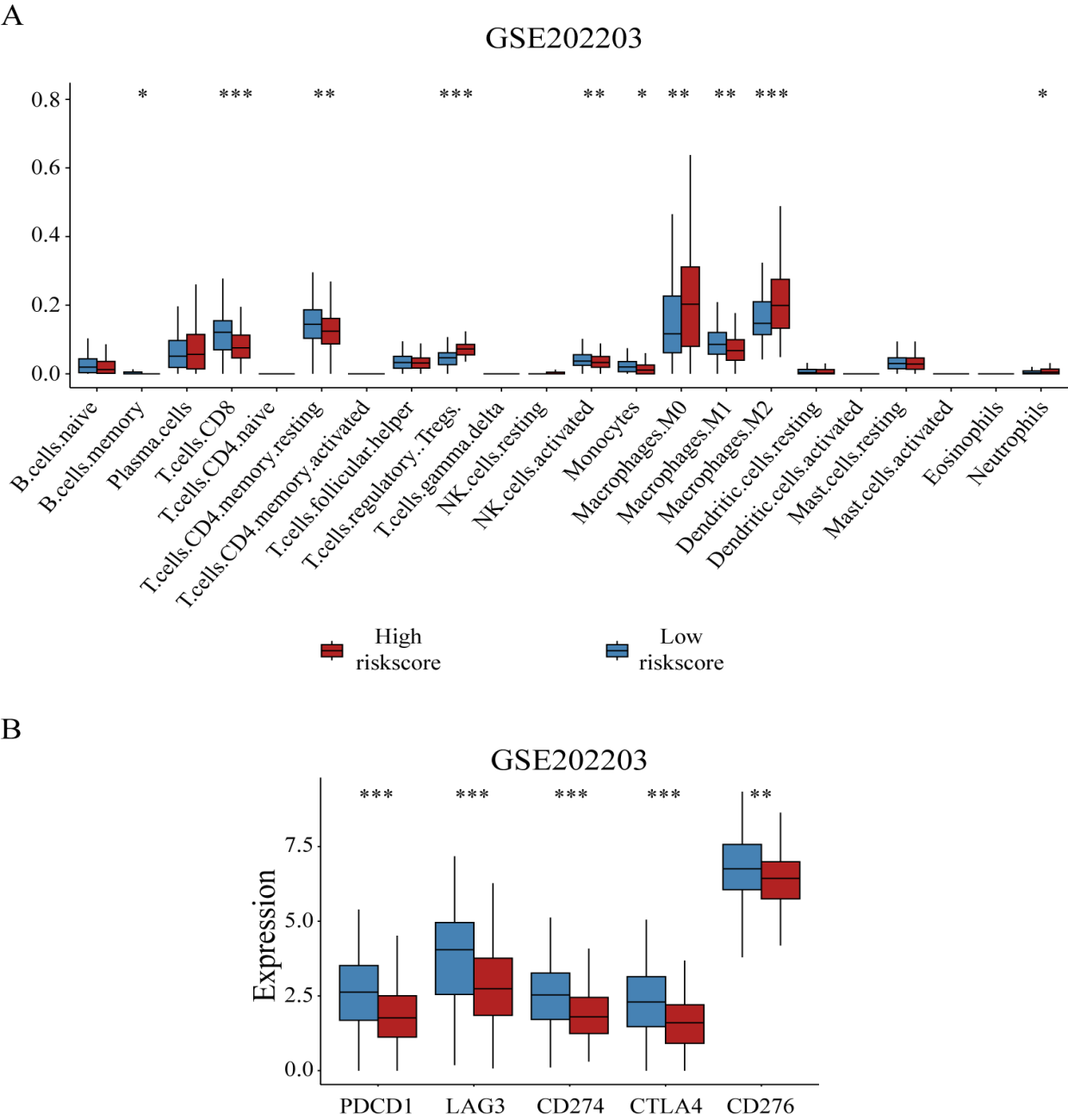


**Supplementary figure S7. Differences in immune-related characteristics between high- and low-risk score groups in GSE202203.** (A) Differences in immune cell infiltration between high- and low-risk score groups. (B) Differences in expression of known immune checkpoint genes between high- and low-risk score groups. ‘*’ indicates p-value ≤ 0.05, ‘**’ indicates p-value ≤ 0.01, ‘***’ indicates p-value ≤ 0.001.
