# Supplementary material for: Regulatory T cells are associated with the tumor immune microenvironment and immunotherapy response in triple-negative breast cancer
Source: Front Immunol. 2023 Sep 12;14:1263537. doi: 10.3389/fimmu.2023.1263537 (PMC10521732; doi:10.3389/fimmu.2023.1263537)

敲减TK1

231

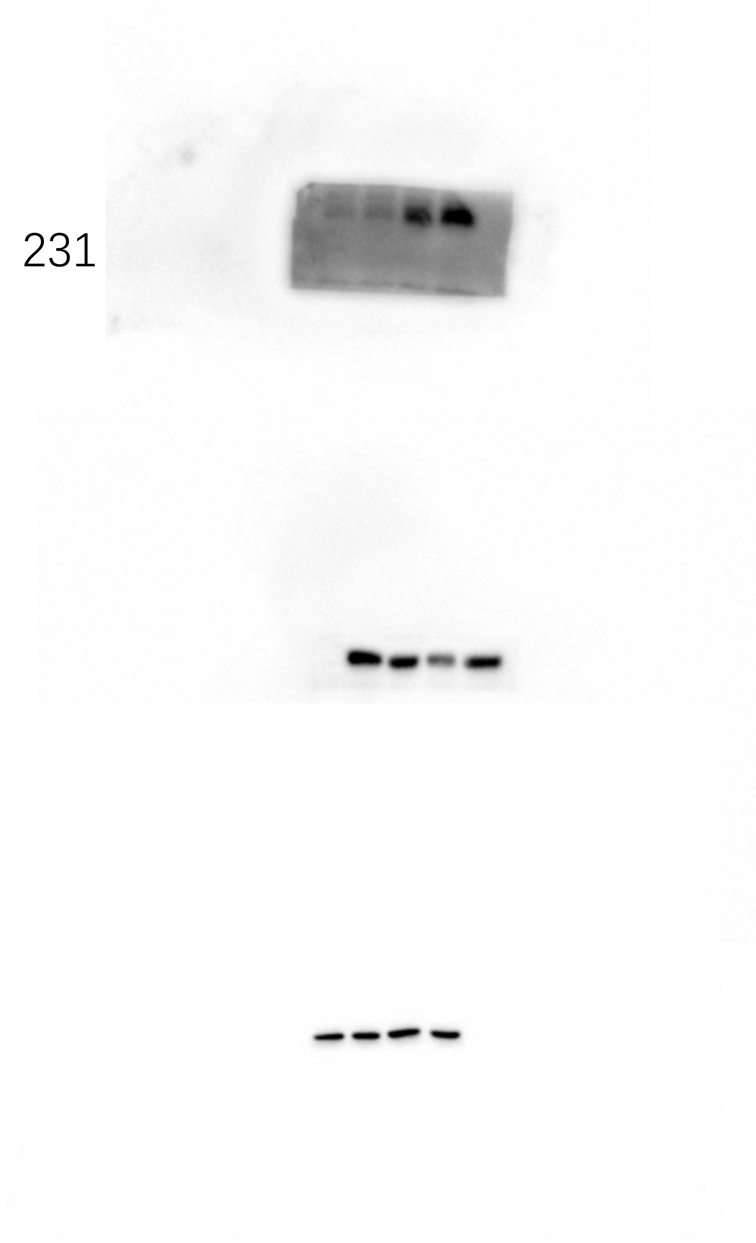

549

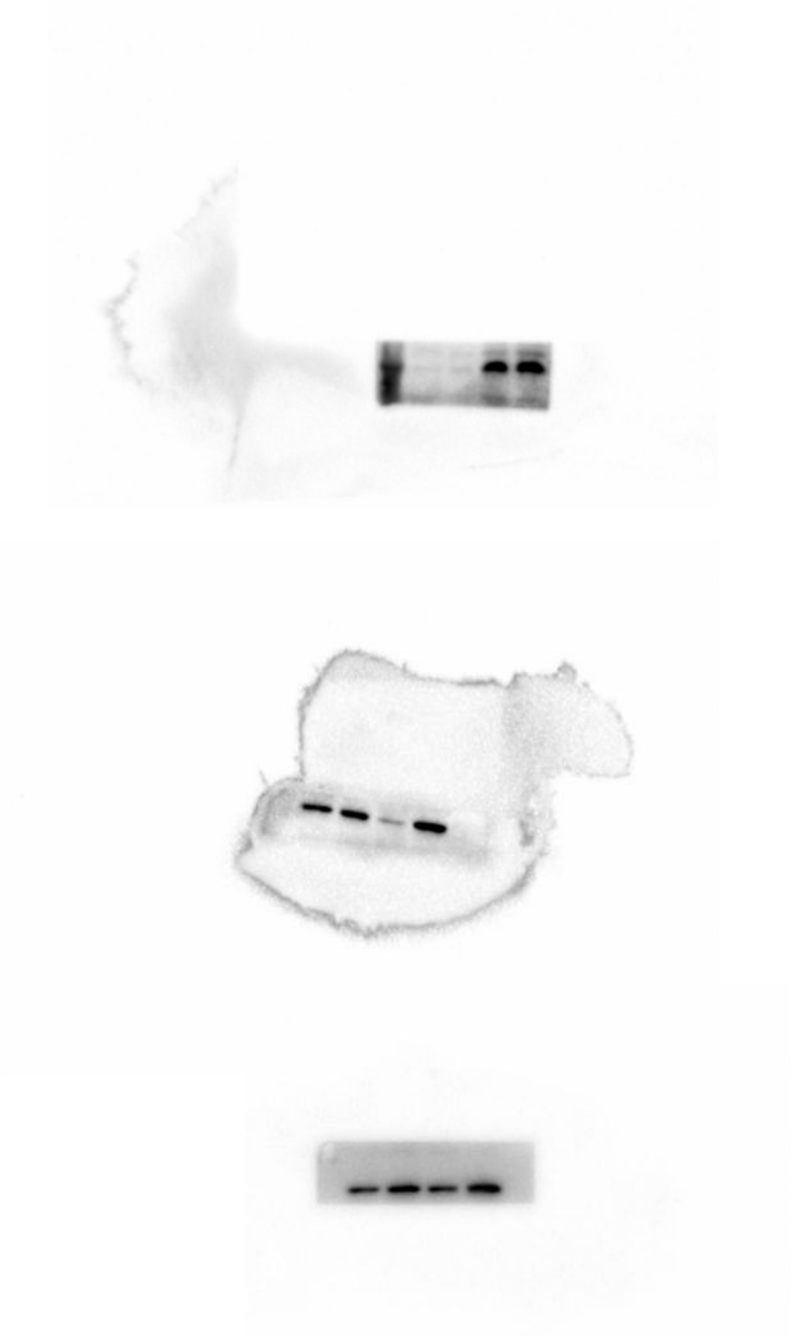

划痕 MDA-MB-231 0h

si-nc

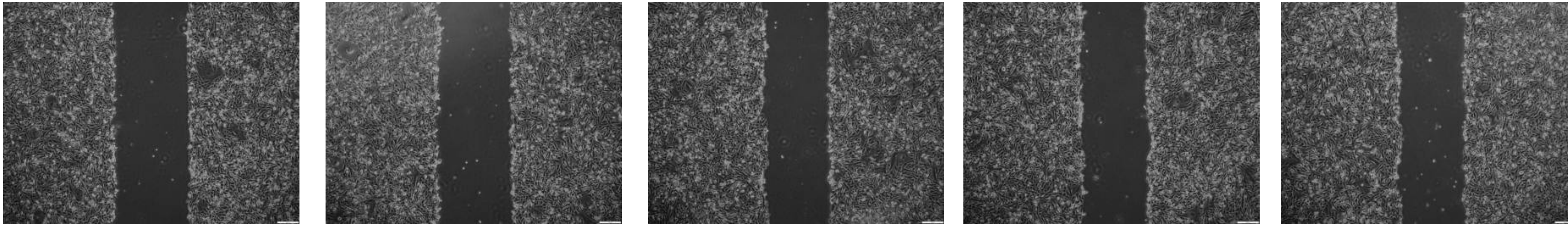

si-1

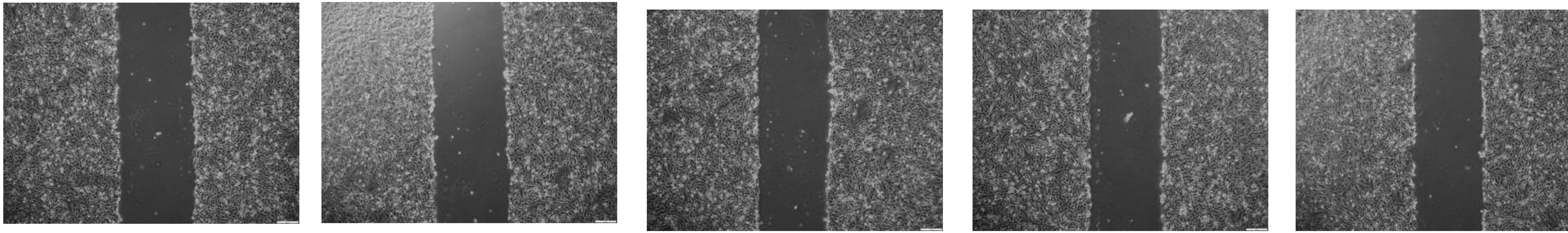

si-2

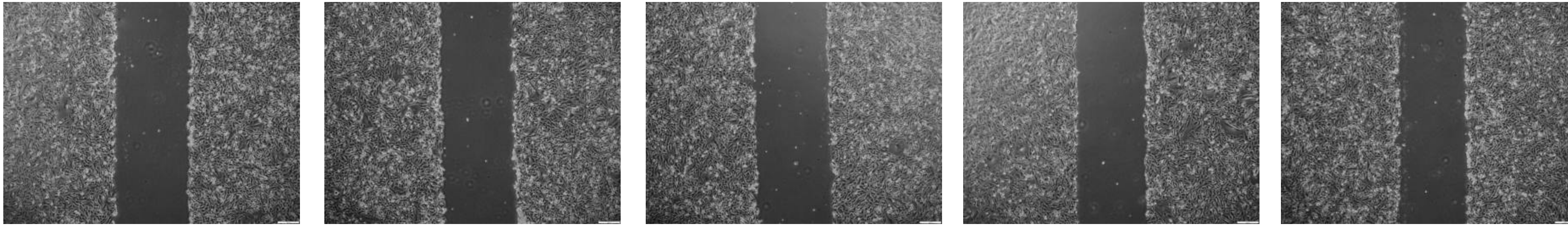

划痕 MDA-MB-231 24h

si-nc

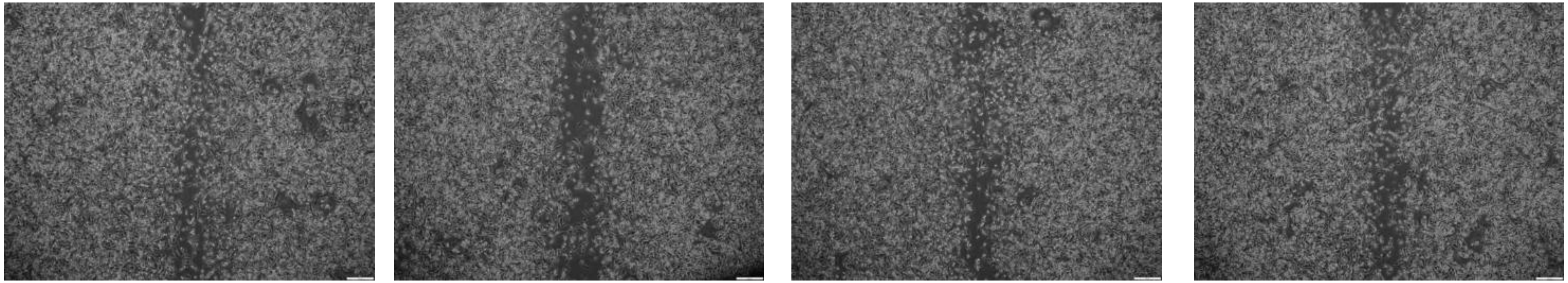

si-1

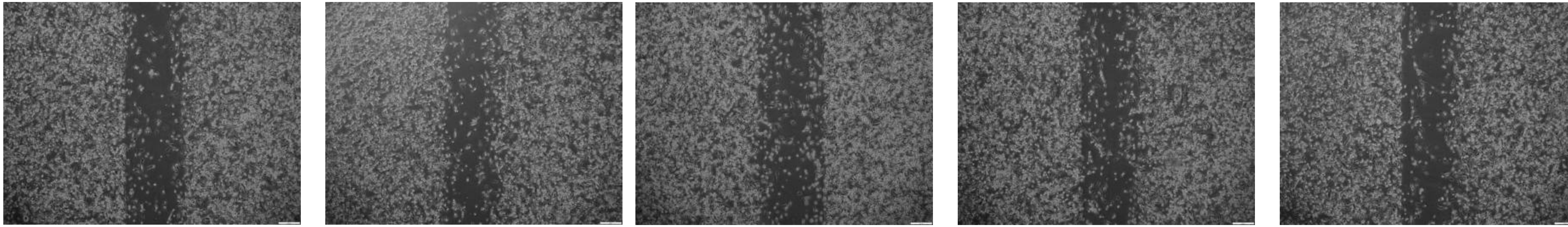

si-2

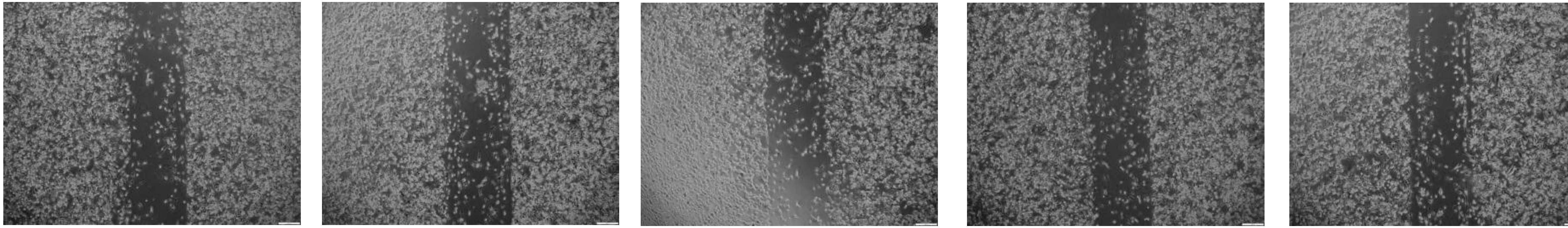

BT-549 0h

si-nc

si-1

si-2

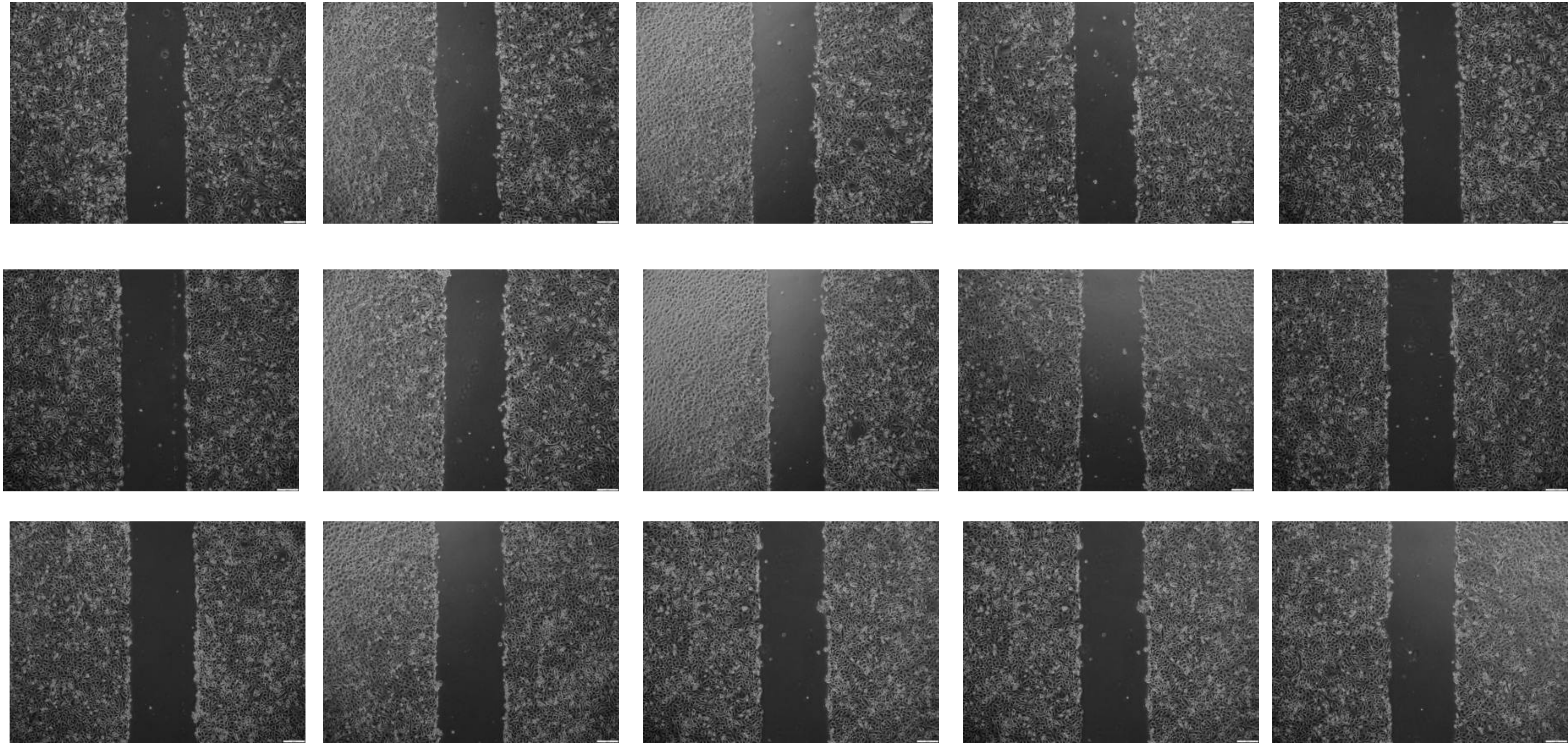

BT-549 24h

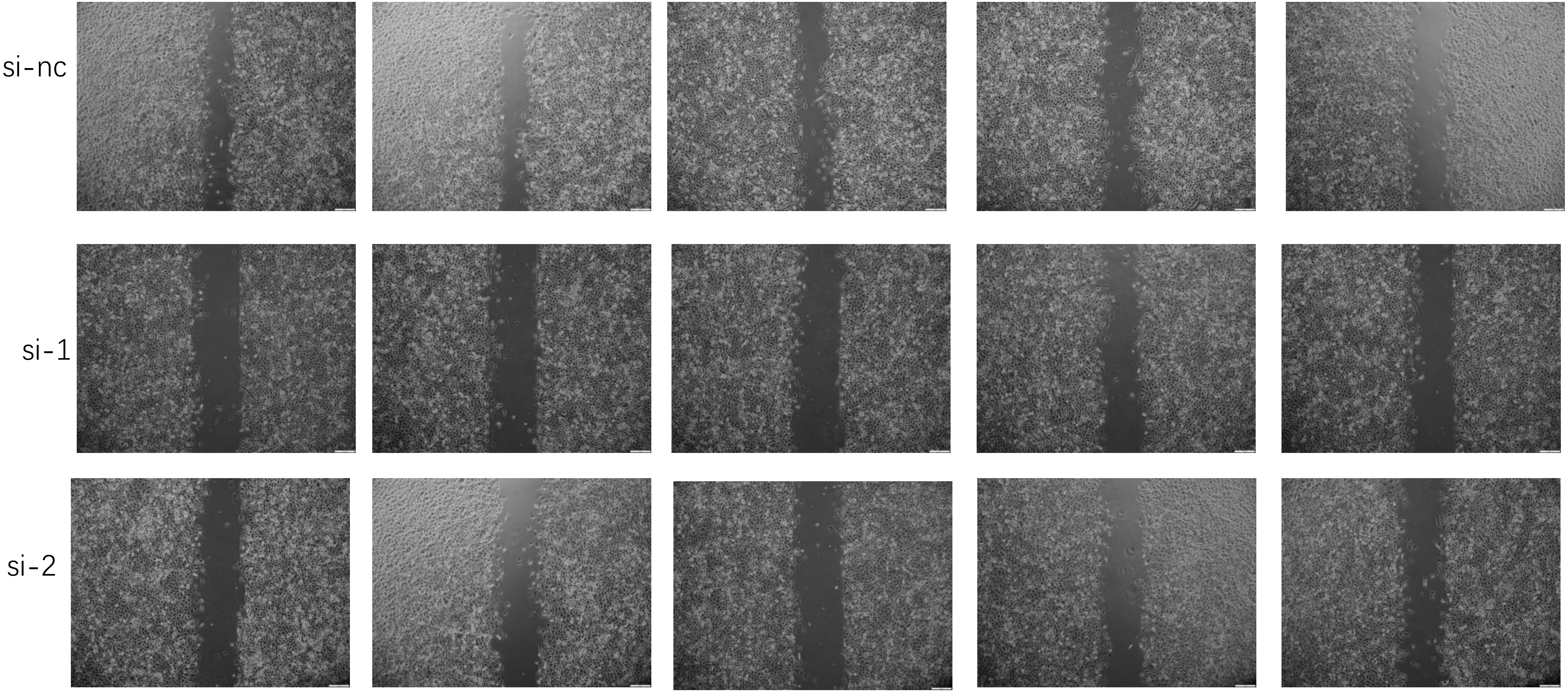

transwell MDA-MB-231

si-nc

si-1

si-2

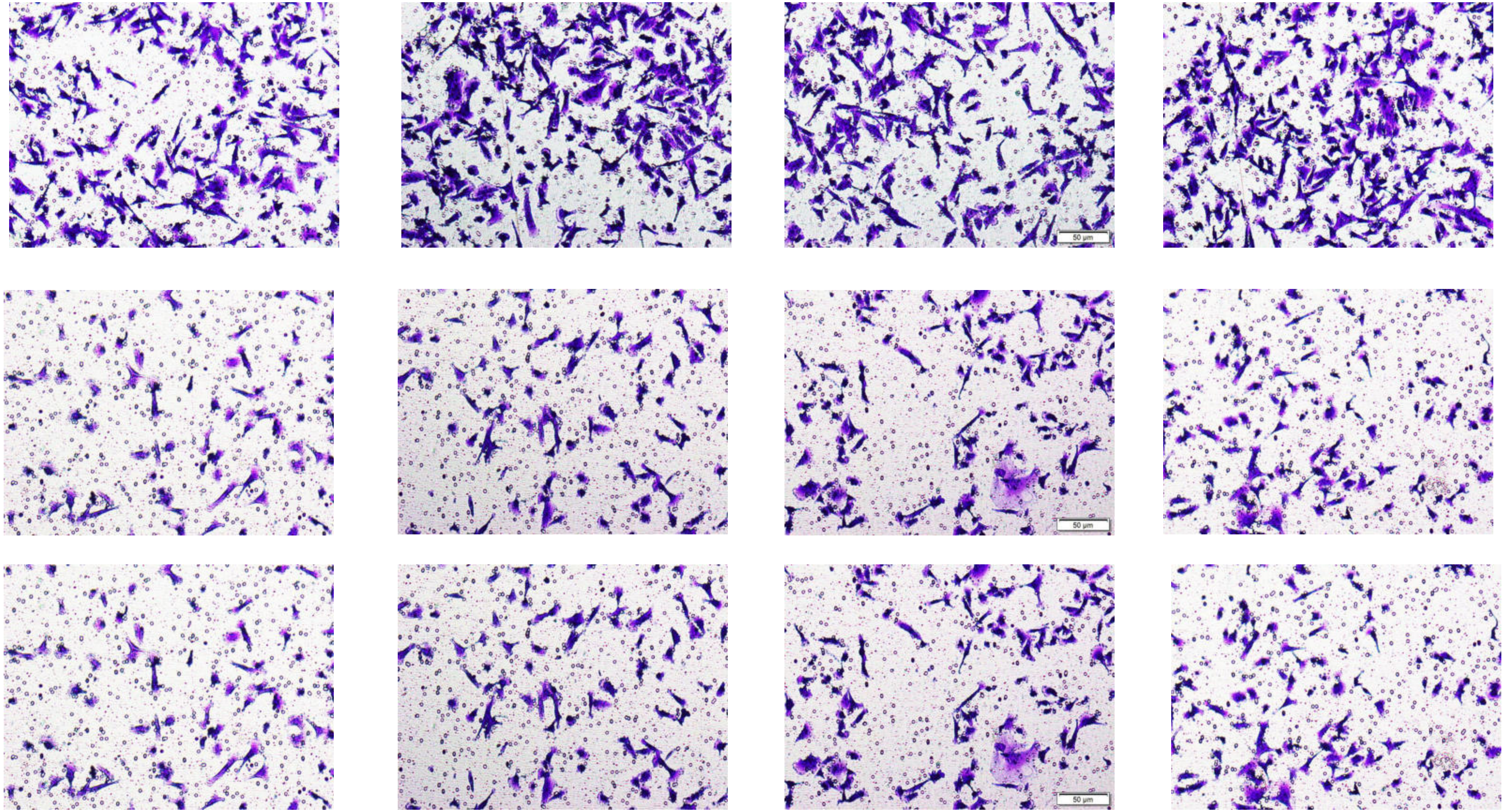

BT-549

si-nc

si-1

si-2

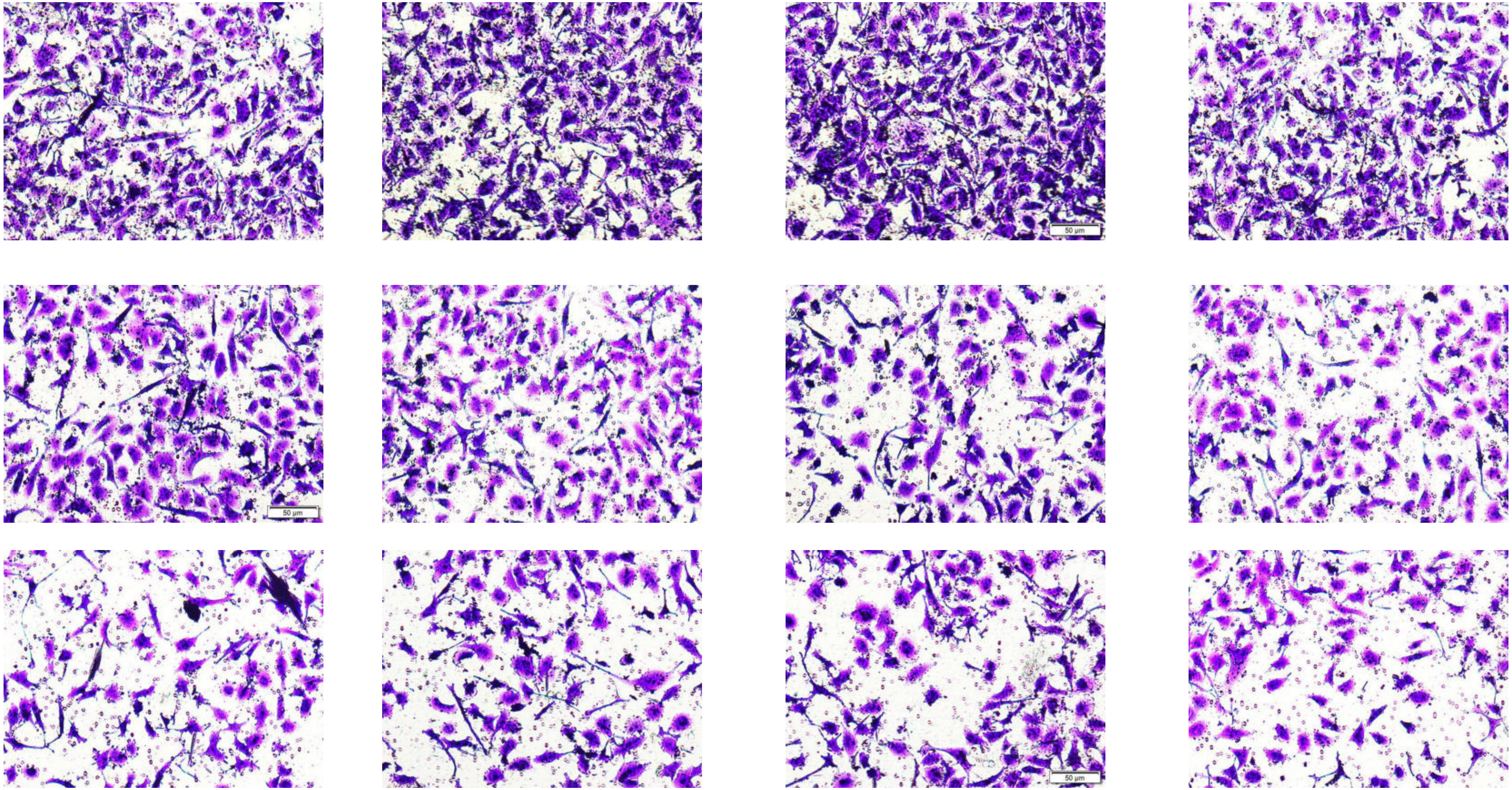

平板克隆

MDA-MB-231 BT-549

si-nc

si-1

si-2

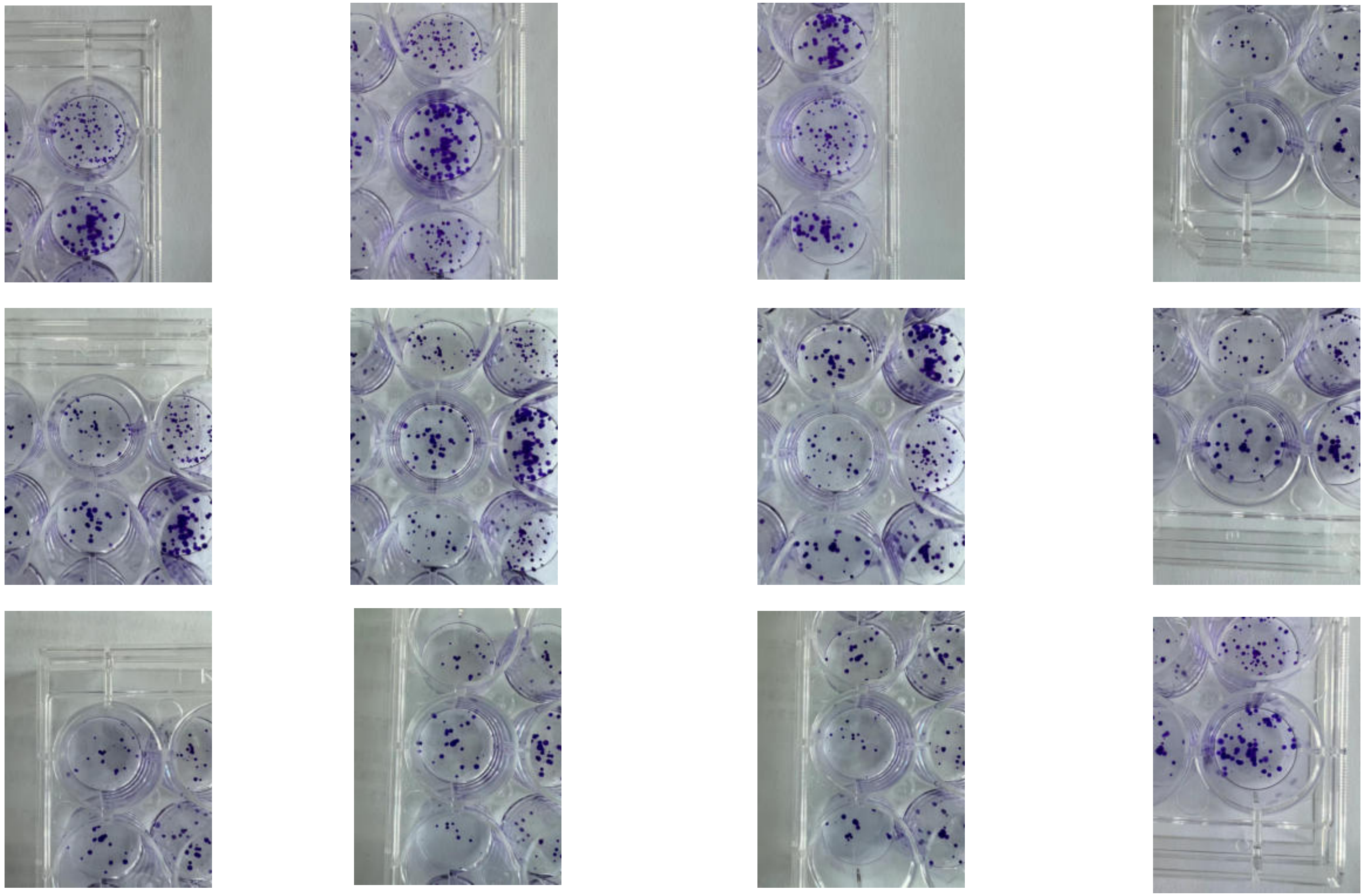

Supplement: Supplementary file 2 [file DataSheet_2.pdf]
