# Supplementary material for: Regulatory T cells are associated with the tumor immune microenvironment and immunotherapy response in triple-negative breast cancer
Source: Front Immunol. 2023 Sep 12;14:1263537. doi: 10.3389/fimmu.2023.1263537 (PMC10521732; doi:10.3389/fimmu.2023.1263537)
Supplement: Supplementary file 3 [file Table_1.docx]

Supplementary table 1. TCGA pan-cancer cohorts.

| Malignant tumors | Abbreviations |
| --- | --- |
| adrenocortical cancer | ACC |
| bladder urothelial carcinoma | BLCA |
| breast invasive carcinoma | BRCA |
| cervical cancer | CESC |
| cholangiocarcinoma | CHOL |
| colon adenocarcinoma | COAD |
| large B-cell lymphoma | DLBC |
| esophageal carcinoma | ESCA |
| glioblastoma multiforme | GBM |
| lower grade glioma and glioblastoma | LGG |
| head and neck squamous cell carcinoma | HNSC |
| kidney chromophobe | KICH |
| kidney renal clear cell carcinoma | KIRC |
| kidney renal papillary cell carcinoma | KIRP |
| acute myeloid leukemia | LAML |
| liver hepatocellular carcinoma | LIHC |
| lung adenocarcinoma | LUAD |
| lung squamous cell carcinoma | LUSC |
| mesothelioma | MESO |
| ovarian cancer | OV |
| pancreatic adenocarcinoma | PAAD |
| pheochromocytoma and paraganglioma | PCPG |
| prostate adenocarcinoma | PRAD |
| rectum adenocarcinoma | READ |
| sarcoma | SARC |
| skin melanoma | SKCM |
| stomach adenocarcinoma | STAD |
| testicular cancer | TGCT |
| thyroid carcinoma | THCA |
| thymoma | THYM |
| uterine corpus endometrial carcinoma | UCEC |
| uterine carcinosarcoma | UCS |
| ocular melanomas | UVM |

Supplementary table 2. Detailed information of the antibodies utilized in this study.

| Antibody | Company (Cat No.) | Dilution (WB) |
| --- | --- | --- |
| TK1 | Proteintech (15691-1-AP) | 1:800 (WB), 1:200 (MIF) |
| β-actin | ZSGB-BIO(TA-09) | 1:2000 |
| GAPDH | Affinity (AF7021) | 1:3000 |
| Foxp3 | Abcam (Ab215206) | 1:200 |

Supplementary table 3. Univariate and multivariate analyses of clinicopathological characteristics and risk score with overall survival in METABRIC and GSE202203 cohorts

| NA | Univariate analysis |  | Multivariate analysis |  |
| --- | --- | --- | --- | --- |
|  | HR (95% CI) | *P* value | HR (95% CI) | *P* value |
| **METABRIC (n=298)** |  |  |  |  |
| Chemotherapy_yes | 1.072(0.784-1.465) | 0.663 |  |  |
| Hormone therapy_yes | 1.412(1.016-1.964) | 0.04 | 1.157(0.771-1.736) | 0.482 |
| Radiotherapy_yes | 0.852(0.607-1.196) | 0.354 |  |  |
| Age | 1.023(1.011-1.035) | < 0.001 | 1.009(0.994-1.025) | 0.228 |
| Tumor Grade | 1.037(0.666-1.614) | 0.872 |  |  |
| Tumor Stage | 2.082(1.271-3.412) | 0.004 | 1.206(0.679-2.14) | 0.523 |
| Tumor size | 1.009(1.003-1.015) | 0.004 | 1.013(1.003-1.024) | 0.014 |
| Risk score | 2.728(2.021-3.682) | < 0.001 | 1.975(1.379-2.828) | < 0.001 |
| **SCAN-B (n = 333)** |  |  |  |  |
| Chemotherapy_yes | 0.299(0.192-0.465) | < 0.001 | 0.596(0.322-1.104) | 0.100 |
| Hormone therapy_yes | 0.408(0.057-2.931) | 0.373 |  |  |
| lymph_node | 1.531(0.942-2.488) | 0.086 |  |  |
| Age | 1.053(1.036-1.071) | < 0.001 | 1.032(1.009-1.055) | 0.006 |
| Tumor Grade | 0.956(0.519-1.763) | 0.886 |  |  |
| Tumor size | 1.028(1.017-1.038) | < 0.001 | 1.014(1.002-1.027) | 0.022 |
| Risk score | 1.721(1.218-  2.43) | < 0.001 | 1.393(1.127-2.001) | 0.007 |
